# Downregulation of Sox8 mediates monosodium urate crystal-induced autophagic impairment of cartilage in gout arthritis

**DOI:** 10.1038/s41420-023-01388-z

**Published:** 2023-03-14

**Authors:** Lu Xiao, Shudian Lin, WenChao Xu, Erwei Sun

**Affiliations:** 1 Department of Rheumatology and Immunology, The Third Affiliated Hospital, Southern Medical University, Guangzhou, China; 2 Department of Rheumatology and Immunology, Hainan General Hospital (Hainan Affiliated Hospital of Hainan Medical University), Hainan, China; 3 Department of Rheumatology and Immunology, Shunde Hospital, Southern Medical University (The First People’s Hospital of Shunde), Jiazi Road, Lunjiao Town, Shunde District, Foshan, 528300 China

**Keywords:** Gout, Inflammation

## Abstract

The deposition of monosodium urate (MSU) crystals in arthritic joints of gout seriously damages cartilage. This study aimed to investigate whether MSU crystal-induced cartilage impairment was related to autophagic signaling. mRNAs of cartilage from MSU-induced gouty arthritis rat model were sequenced. MSU crystal-treated human chondrocytes were used to evaluate the function of Sox8. The recombinant Sox8 lentiviral vector (lenti-Sox8) was applied to upregulate the expression of Sox8. Transfection of the mRFP-GFP-LC3 plasmid was evaluated by confocal microscopy. The autophagic vacuoles were stained with monodansylcadaverine and examined by flow cytometry. The morphology of autophagosomes was observed by transmission electron microscopy. The ratio of LC3-II/I in the presence or absence of bafilomycin A1 and the expression levels of Beclin1, Sox8, p-PI3K, PI3K, p-AKT, AKT, p-mTOR, and mTOR were detected by Western blot. In vivo, the effect of Sox8 on cartilage of acute gouty model rats was evaluated by safranin-O/fast green staining and Western blot. The expression of Sox8 was significantly downregulated both in vivo and in vitro. In chondrocytes, MSU crystals reduced the expression of Sox8, inhibited the PI3K/AKT/mTOR signaling pathway, and increased the level of autophagy. Overexpression of Sox8 notably inhibited MSU crystal-induced autophagy by rescuing the phosphorylation levels in the PI3K/AKT/mTOR signaling pathway. In vivo, overexpression of Sox8 remarkably alleviated cartilage damage in acute gouty model rats. These results indicate that downregulation of Sox8 plays an important role in MSU-induced chondrocyte autophagy by modulating PI3K/AKT/mTOR signaling, and overexpression of Sox8 may serve as a novel therapy to prevent the impairment of cartilage in gout arthritis.

## Introduction

Gout arthritis, the most common inflammatory arthropathy in young men, is characterized by the deposition of monosodium urate (MSU) crystals in joints [1]. Subjects with high MSU levels have focal erosions, osteophytes, and bone marrow lesions [2]. Cartilage injury is a serious complication of gout arthritis and MSU crystals have profound inhibitory effects on chondrocyte viability and function [3]. Large scaled studies have revealed strong correlations between the deposition of MSU crystals and cartilage lesions [4, 5]. However, the mechanism of MSU crystals on chondrocytes injury remains unclear. Autophagy is an intracellular degradation system that is mediated by PI3K/AKT pathway and mTOR [6–9]. Interestingly, it has been discovered that MSU crystals activate autophagy and induce cell death via the inhibition of phosphorylation of AKT/mTOR signaling pathway [10]. However, the key molecules mediating chondrocyte autophagic cell death in acute gout arthritis have not been identified.

The SOX family proteins include 20 different subtypes (termed from A to H), and Sox8 belongs to the E subgroup [11]. Sox8 usually acts as a transcriptional activator after forming protein complexes with other proteins [12] and encodes a member of the SOX family of transcription factors involved in the determination of cell fate and in the regulation of embryonic development [13].

In this study, we found that the downregulation of Sox8 contributed to MSU-induced cartilage autophagic cell death by inhibition of PI3K/AKT/mTOR pathway, and overexpression of Sox8 markedly alleviated MSU-induced cartilage damage in gout arthritis. The overall flowchart of this study is shown in Supplementary Fig. 1.

## Results

### The mRNA expression of Sox8 was significantly downregulated in gout arthritis model rats and MSU crystal-treated chondrocytes

**Fig. 1:**
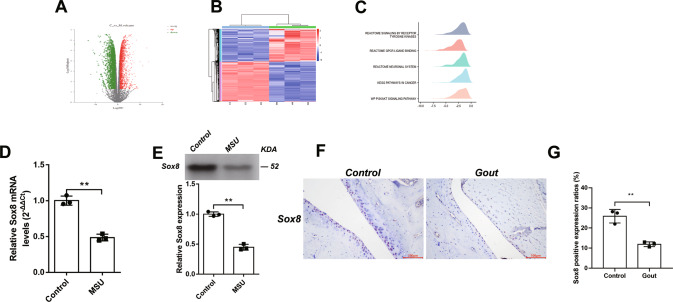
The mRNA expression of Sox8 was significantly downregulated in gouty arthritis rat models and MSU crystal-treated chondrocytes. Volcano plot (**A**) and heatmap (**B**) of the mRNA sequencing result of cartilage from control group and gouty arthritis models. In the volcano plot, the green ones stand for downregulated genes and the red ones for upregulated genes; C and M in plots stands for control and model (gouty arthritis models) group respectively. In the heatmap, the blue ones represent downregulated genes and the red ones for upregulated genes. GSEA (**C**) illustrated the differentially identified genes. C28/I2 cells were incubated with MSU (200 μg/mL) for 24 h and Sox8 mRNA determined. The expression of Sox8 mRNA was significantly decreased both at mRNA level (**D**) and at protein level (**E**). The protein expression of Sox8 (**F** and **G**) in the cartilage of gouty arthritis rat models and the controls. Cartilage was stained with blue and Sox8 was stained yellow.

High-quality sequencing of mRNA from rat cartilage was performed with an Illumina Novaseq 6000 device. Specific criteria (Adjusted *P* value < 0.05 and |log2 fold change|>2) were set as the threshold and considered significant between the control and acute gouty model. The volcano plot and heatmap are shown in Fig. 1A, B. Gene set enrichment analysis (GSEA) was carried out to identify the possible pathological processes. The top three negatively enriched gene sets were PI3K/AKT signaling pathway, KEGG pathway in cancer, and Reactome neuronal system in GSEA (Fig. 1C). Importantly, the expression of Sox8 mRNA and protein significantly decreased after MSU crystal treatment (Fig. 1D, E). In addition, the protein expression of Sox8 was significantly downregulated in the gouty rats (Fig. 1F, G).

### MSU crystal-activated autophagy

**Fig. 2:**
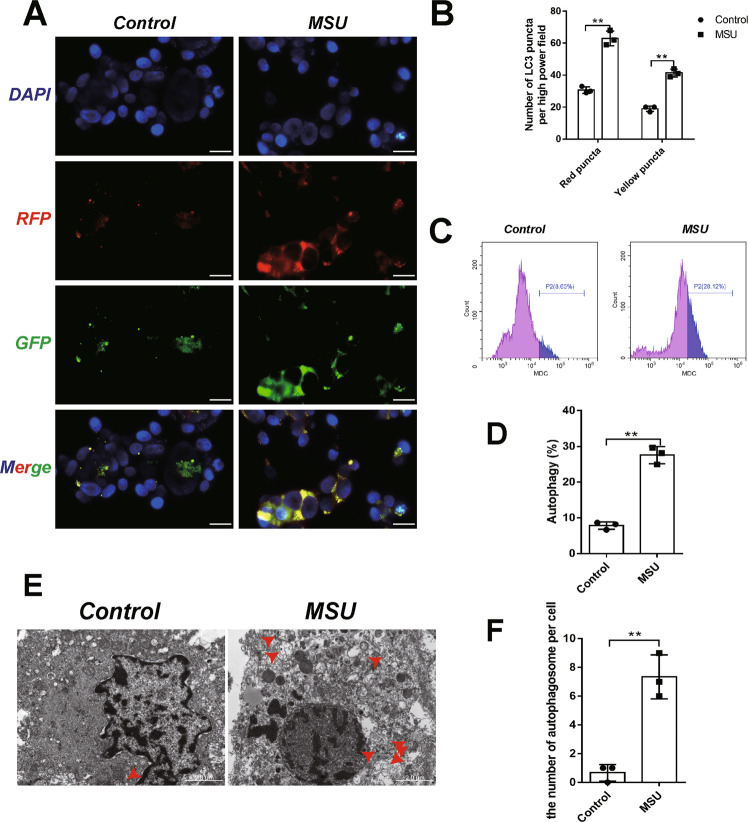
MSU-induced autophagy of C28/I2 cells. C28/I2 cells were incubated with MSU (200 μg/mL) for 24 h. Transfection of the mRFP-GFP-LC3 (**A**, **B**) plasmid into C28/I2 cells and quantification of the autophagic puncta by confocal microscopy, bar = 20 μm. To evaluate autophagic vacuoles, C28/I2 cells were stained with MDC, and the autophagy ratio of C28/I2 cells in the indicated groups was measured by flow cytometry (**C**, **D**). The morphology (**E**) and the number (**F**) of autophagosome in C28/I2 cells were observed by transmission electron microscope, bar = 2 μm. The red arrow points to the autophagosome. Results are the mean ± SD for three individual experiments. *P < 0.05, **P < 0.01.

As MSU crystal has been found to induce autophagy in human chondrocytes, our findings that MSU crystal downregulated Sox8 expression prompted us to assume that Sox8 might play a key role in MSU-induced autophagy in chondrocytes. In order to verify this, we first examined the effect of MSU crystals on autophagy induction in C28/I2 chondrocytes transfected with mRFP-GFP-LC3 adenovirus. As shown in Fig. 2A, B, the numbers of yellow and red puncta increased remarkably in MSU-treated cells. Autophagic vacuoles stained with Monodansylcadaverine (MDC), a fluorescent dye that labels late-stage autophagosomes, increased in MSU-treated cells (Fig. 2C, D). Furthermore, the increased autophagy activity in MSU crystal-treated cells was verified by ultra-structural analysis of autophagosomes using a transmission electron microscope (TEM), showing that C28/I2 cells exposed to MSU crystals for 24 h displayed an increase in the autophagy-related vesicles (Fig. 2E, F, red arrow). Therefore, our results confirmed that MSU treatment significantly increased autophagy in C28/I2 cells.

**Fig. 3:**
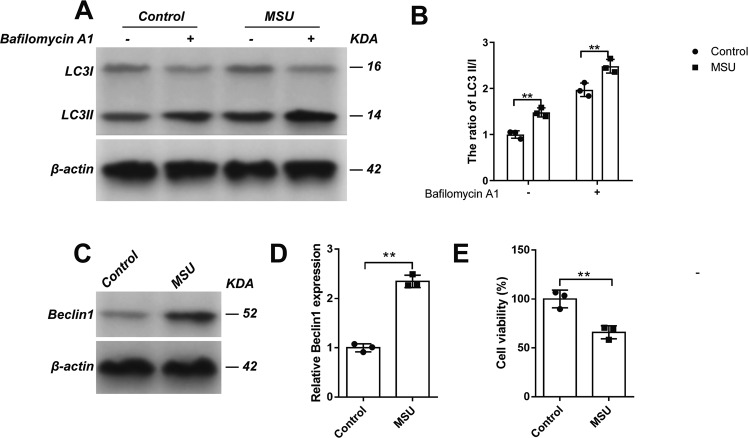
MSU induced the autophagy of C28/I2 cells. C28/I2 cells were incubated with MSU (200 μg/mL) for 24 h. The ratio of LC3-II/I (**A**, **B**) in the presence or absence of bafilomycin A1 was detected by western blot assay. The expression of Beclin1 (**C**, **D**) in the indicated groups was detected by western blot assay. Cell viability (**E**) was detected by CCK-8 assay. Results are the mean ± SD for three individual experiments. *P < 0.05, **P < 0.01.

During the autophagy, LC3-I (16 kDa) is changed to LC3-II (14 kDa), a double-membrane structure, which makes the ratio of LC3II/I as a unique characteristic for autophagy. As the generation of LC3-II and degradation of LC3-I occur continuously during autophagy, the expression of LC3-I and LC3-II at a single time point cannot reflect the change of autophagy. Therefore, autophagy flux that more precisely represents autophagy activity is frequently measured by using drugs (such as Bafilomycin A1) to block lysosomal degradation of autophagosomes [14]. To this end, cells with or without MSU crystals were treated with or without Bafilomycin A1 (10 nM) for 24 h and then autophagy activity was examined. As shown in Fig. 3, the ratio of LC3-II/I markedly increased in MSU-treated cells (Fig. 3A, B). Autophagy flux analysis of LC3-II/I in bafilomycin A1 treated cells confirmed the similar induction of canonical autophagy (Fig. 3B). Interestingly, the expression of Beclin1 was also dramatically elevated after MSU crystal treatment (Fig. 3C, D). Beclin1, the first described mammalian autophagy protein, is a central regulator of autophagy and functions through interaction with the PI3K VPS34, VPS15, and the autophagy protein ATG14 in the initial stages of autophagic vesicle formation [15].In addition, C28/I2 cell viability was also reduced after MSU crystals treatment (Fig. 3E). Taken together, these findings suggested that autophagy pathway was significantly initiated in MSU crystal-treated C28/I2 cells.

### MSU crystals inhibited the PI3K/AKT/mTOR signaling pathway

**Fig. 4:**
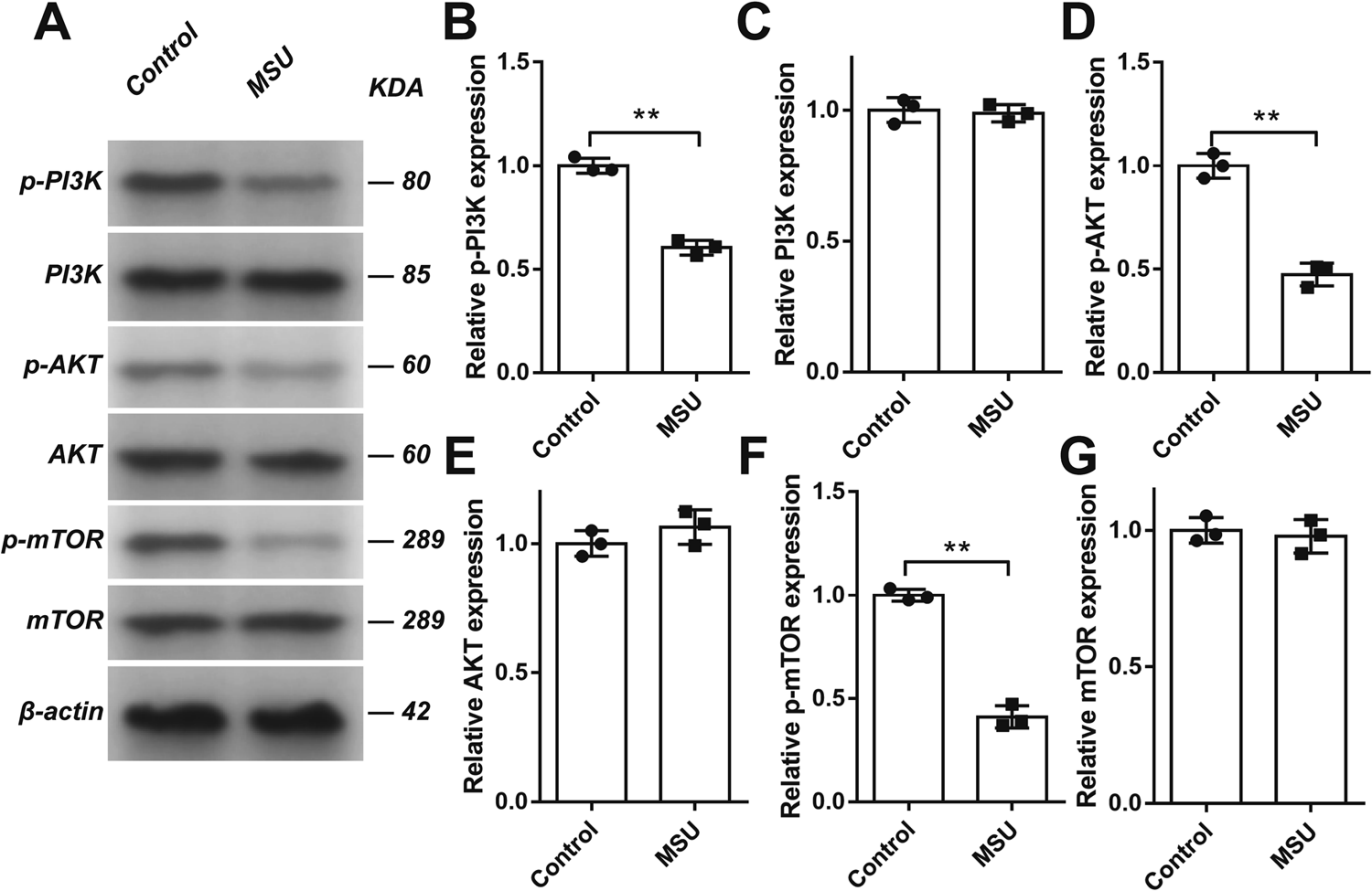
MSU inhibited the PI3K/AKT/mTOR signaling pathway. C28/I2 cells were incubated with MSU (200 μg/mL) for 24 h. The expression of p-PI3K (**A**, **B**), PI3K (**A**, **C**), p-AKT (**A**, **D**), AKT (**A**, **E**), p-mTOR (**A**, **F**), and mTOR (**A**, **G**) was detected by western blot assay. Results are the mean ± SD for three individual experiments. *P < 0.05, **P < 0.01.

The negatively enriched PI3K/AKT signaling genes in gout arthritis models (Fig. 1C) and the involvement of Beclin1 in MSU crystal-induced autophagy of C28/I2 chondrocytes (Fig. 3C) suggested that PI3K signaling played an important role in this process. Therefore, we next investigated whether MSU crystals could inhibit PI3K/AKT/mTOR signaling pathway. C28/I2 cells were incubated with MSU crystals (200 μg/mL) for 24 h and the expressions of p-PI3K, PI3K, p-AKT, AKT, p-mTOR, and mTOR examined with western blot. As shown in Fig. 4, the expressions of p-PI3K (Fig. 4A, B), p-AKT (Fig. 4D), and p-mTOR (Fig. 4A, F) were significantly decreased. These results demonstrated that MSU crystals initiated the autophagy signaling via inhibiting the phosphorylation of the PI3K/AKT/mTOR signaling pathway.

### Sox8 mediated MSU crystal-induced autophagy by rescuing the phosphorylation in PI3K/AKT/mTOR signaling pathway

**Fig. 5:**
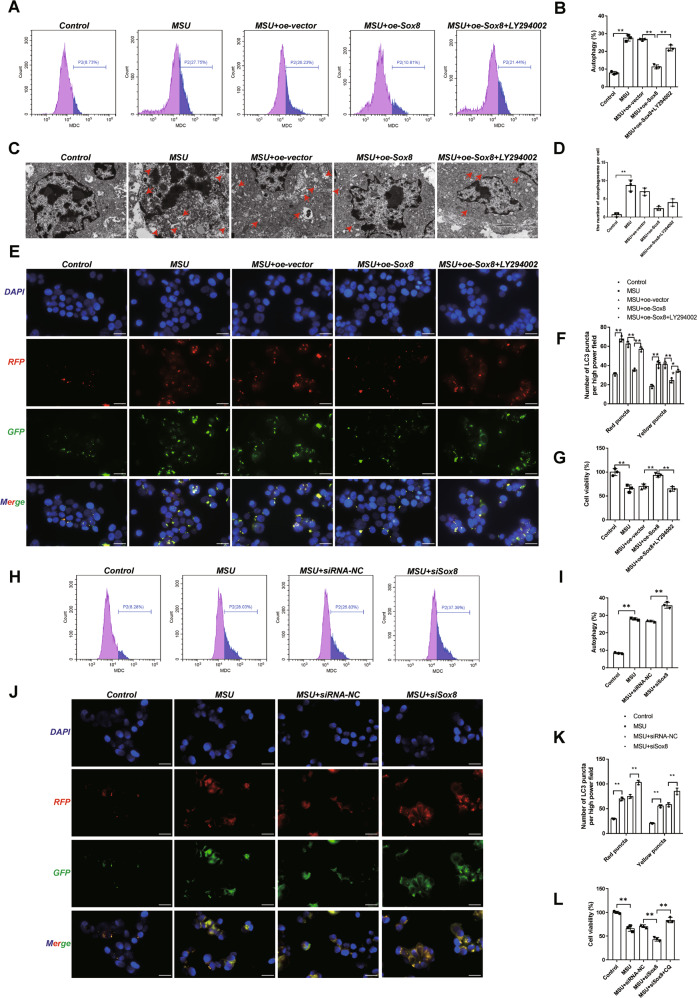
Sox8 mediated the autophagy induced by MSU via the PI3K/AKT/mTOR signaling pathway. Overexpression of Sox8 rescued the autophagy induced by MSU via the PI3K/AKT/mTOR signaling pathway. C28/I2 cells were transfected with oe-vector or oe-Sox8, then treated with MSU (200 μg/mL) for 24 h and stimulated with LY294002 (10 μM) for 1 h. To evaluate autophagic vacuoles, C28/I2 cells were stained with MDC, and the autophagy ratio of C28/I2 cells in the indicated groups was measured by flow cytometry (**A**, **B**). The morphology (**C**) and the number (**D**) of autophagosomes in C28/I2 cells was observed by transmission electron microscope, bar = 2 μm. The red arrow points to the autophagosome. Transfection of the mRFP-GFP-LC3 (**E**, **F**) plasmid into C28/I2 cells and quantification of the autophagic puncta by confocal microscopy, bar = 20 μm. Cell viability (**G**) was detected by CCK-8 assay. Silencing of Sox8 increased the autophagy induced by MSU. The C28/I2 cells were transfected with siRNA-NC or siSox8, then they were treated with MSU (200 μg/ml) for 24 h. To evaluate autophagic vacuoles, C28/I2 cells were stained with MDC, and the autophagy ratio of C28/I2 cells in the indicated groups were measured by flow cytometric analysis (**H**, **I**). Transfection of the mRFP-GFP-LC3 (**J**, **K**) plasmid into C28/I2 cells and quantification of the autophagic puncta by confocal microscopy, bar = 20 μm. The C28/I2 cells were transfected with siRNA-NC or siSox8, then they were treated with MSU (200 μg/ml) for 24 h or incubated with MSU (200 μg/ml) or MSU (200 μg/ml) +CQ (20 μM) for 24 h. The cell viability (**L**) was detected by CCK-8 assay. Results are the mean ± SD for three individual experiments. *P < 0.05, **P < 0.01.

As abovementioned, MSU crystals activated autophagy and inhibited PI3K/AKT/mTOR signaling pathway and Sox8 was significantly downregulated after MSU crystal treatment both in vivo and in vitro. Therefore, we hypothesized a possible link between Sox8 and MSU crystal-induced autophagy, as well as the important role of PI3K/AKT/mTOR signaling pathway in this process. To verify this, C28/I2 cells were first transfected with vector or oe-Sox8, incubated with MSU crystals (200 μg/ml) for 24 h, and then treated with a PI3K inhibitor, LY294002 (10 μM), for 1 h. LY294002 was the first synthesized small molecule known to inhibit PI3Kα/δ/β. As shown in Fig. 5A, B, according to the results from MDC staining, the increased autophagy in the MSU group could be blocked by overexpression of Sox8. Interestingly, the effect of Sox8 could be reversed by the inhibition of PI3K signaling. The results were further confirmed by the number of autophagosomes in C28/I2 cells examined with TEM (Fig. 5C, D, red arrow represent autophagosome vesicles) and the autophagic flux detection (Fig. 5E, F). In addition, CCK-8 results showed that the cell activity was significantly decreased after MSU treatment, and overexpression of Sox8 could improve the cell viability, while decreased after the treatment of PI3K inhibitor LY294002 (Fig. 5G).

In addition, the silencing of Sox8 with small interfering RNA (siSox8) in C28/I2 was used to demonstrate the autophagic response of MSU-treated chondrocytes. It is found that autophagy increased more significantly after siSox8 treatment compared with MSU-treated chondrocytes according to MDC and autophagic flux results (Fig. 5H–K). Moreover, cell viability of C28/I2 increased dramatically in MSU + siSox8+ Chloroquine (CQ, an autophagy inhibitor) group compared with that in MSU + siSox8 group (Fig. 5L).

**Fig. 6:**
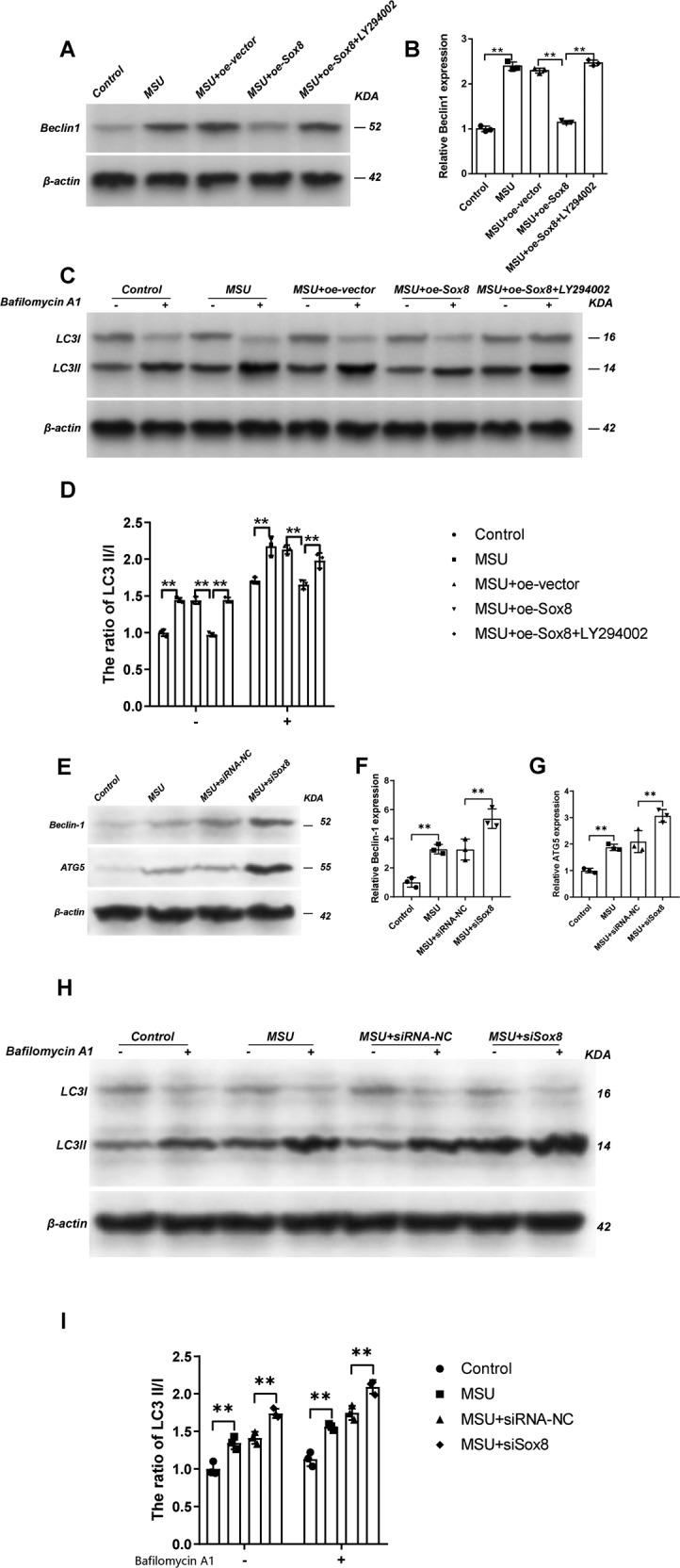
Sox8 mediated the autophagy induced by MSU evaluated by Western blot assay. Overexpression of Sox8 inhibited the autophagy induced by MSU evaluated by Western blot assay. The C28/I2 cells were transfected with oe-vector or oe-Sox8, treated with MSU (200 μg/mL) for 24 h, and stimulated with LY294002 (10 μM) for 1 h. The expression of Beclin1 (**A**, **B**) from the indicated groups was detected by Western blot assay. The ratio of LC3-II/I (**C**, **D**) in the presence or absence of bafilomycin A1 was detected by Western blot assay. Sox8 knockdown increased the autophagy of C28/I2 cells induced by MSU. The C28/I2 cells were transfected with siRNA-NC or siSox8, then they were treated with MSU (200 μg/ml) for 24 h. The expression of Beclin1 (**E**, **F**) and ATG5 (**E**, **G**) from the indicated groups were detected by western blot assay. The ratio of LC3-II/I (**H**, **I**) in the presence or absence of bafilomycin A1 was detected by western blot assay. Results are the mean ± SD for three individual experiments. *P < 0.05, **P < 0.01.

**Fig. 7:**
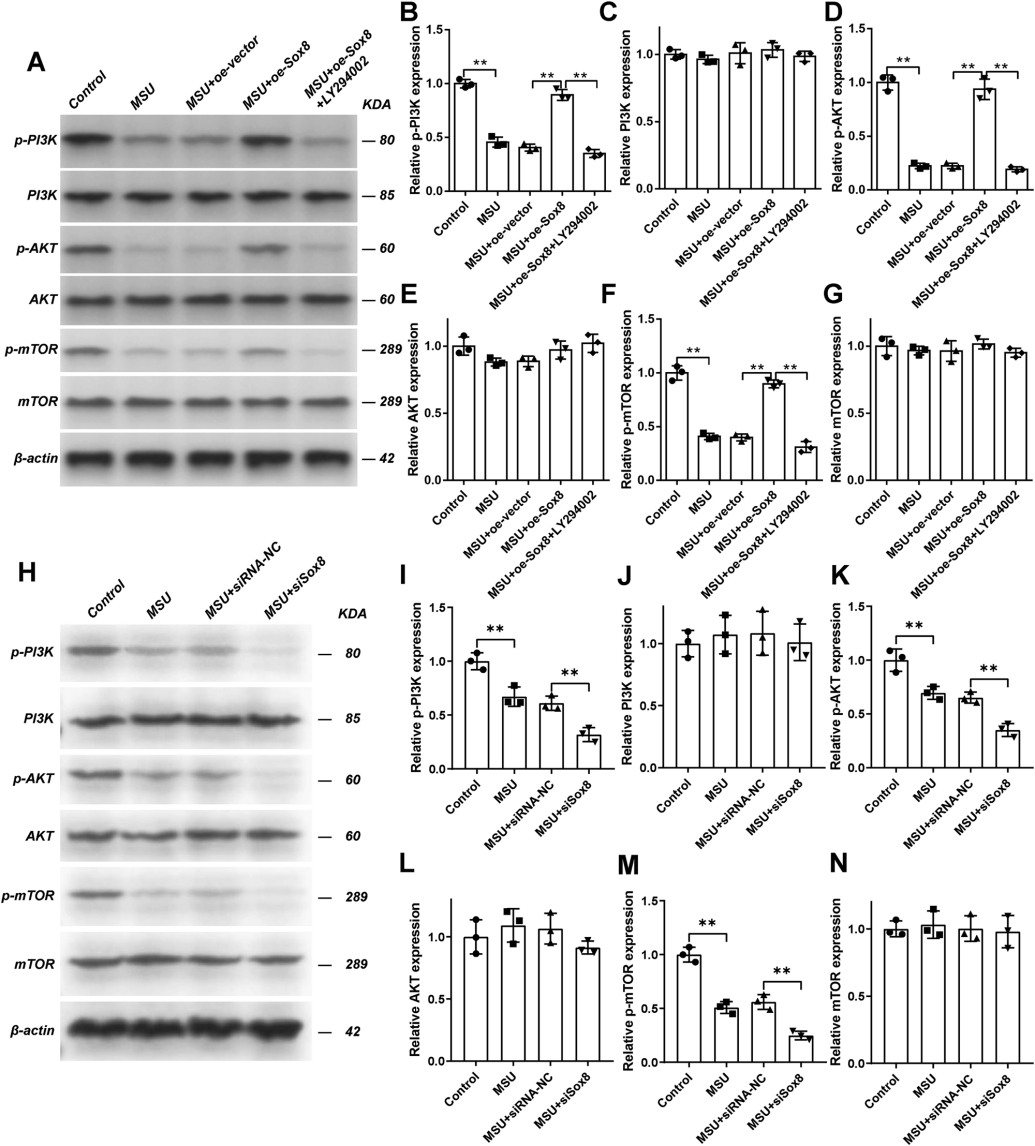
Sox8 mediated the effects of MSU on the PI3K/AKT/mTOR signaling pathway. Overexpression of Sox8 rescued the effects of MSU on the PI3K/AKT/mTOR signaling pathway. The C28/I2 cells were transfected with oe-vector or oe-Sox8 by lentiviral vector, treated with MSU (200 μg/mL) for 24 h, and stimulated with LY294002 (10 μM) for 1 h. The expression levels of p-PI3K (**A**, **B**), PI3K (**A**, **C**), p-AKT (**A**, **D**), AKT (**A**, **E**), p-mTOR (**A**, **F**), and mTOR (**A**, **G**) from the indicated groups were detected by western blot assay. Sox8 knockdown inhibited PI3K/AKT/mTOR signaling pathway. The C28/I2 cells were transfected with siRNA-NC or siSox8, then they were treated with MSU (200 μg/ml) for 24 h. The expression of p-PI3K (**H**, **I**), PI3K (**H**, **J**), p-AKT (**H**, **K**), AKT (**H**, **L**), p-mTOR (**H**, **M**), mTOR (**H**, **N**) from the indicated group were detected by western blot assay. Results are the mean ± SD for three individual experiments. *P < 0.05, **P < 0.01.

In order to further examine the effect of Sox8, the expression of autophagic marker protein, including Beclin1 and LC3II/I was detected. The lysosomotropic reagent Bafilomycin A1 (10 nM) was used to analyze the autophagy flux. The results demonstrated that oe-Sox8 could statistically decrease the expression of Beclin1 (Fig. 6A, B) and the ratio of LC3-II/I (Fig. 6C, D), whereas the inhibitor of PI3K, LY294002, blocked the effect of overexpressed Sox8. Moreover, when Sox8 was silenced, the levels of Becilin1, ATG5, and LC3 II/I were all increased more comparing solely MSU-treated chondrocytes (Fig. 6E–I). To further establish the effect of Sox8 on PI3K/AKT/mTOR signaling, the phosphorylation of the molecules was examined. The results demonstrated that overexpression of Sox8 could increase the level of phosphorylation of PI3K (Fig. 7A–C), AKT (Fig. 7A, D, E), and mTOR (Fig. 7A, F, G), and LY294002 rescued the effect of Sox8. Whereas siSox8 could decrease the phosphorylation of PI3K (Fig. 7H, I, J), AKT (Fig. 7H, K, L), and mTOR (Fig. 7H, M, N) more as compared with MSU-treated chondrocytes (Fig. 7H–N). Taken together, these results indicate that overexpression of Sox8 ameliorated MSU crystal-induced autophagy via inhibiting the phosphorylaton of PI3K/AKT/mTOR signaling pathway.

### Sox8 overexpression alleviated the cartilage damage of MSU crystal-induced gouty arthritis rats

**Fig. 8:**
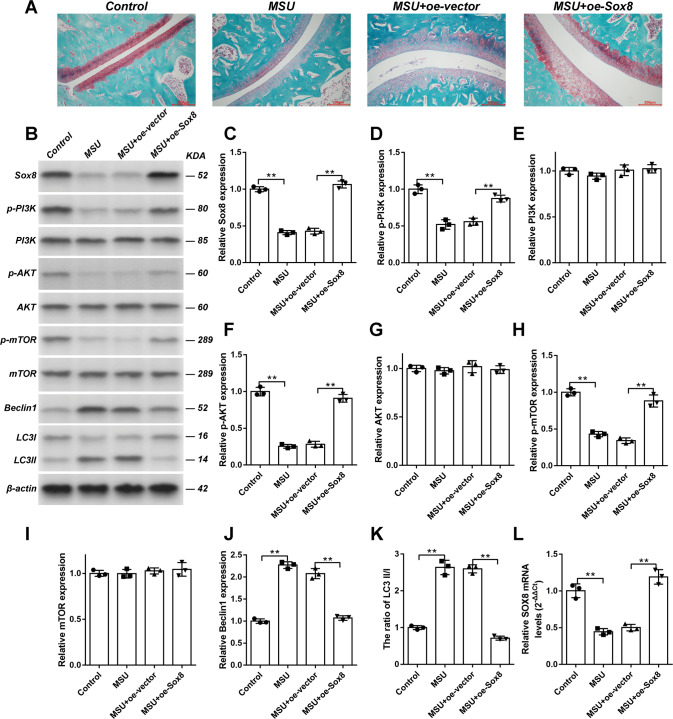
Effect of Sox8 overexpression on cartilage and PI3K/AKT/mTOR signaling pathway in MSU-induced gouty arthritis rat model. The right ankle joints of the rats were injected with oe-vector or oe-Sox8. Followed by, the injection of 100 μL of MSU (30 mg/mL) the next day. The rats were sacrificed on day 3 after MSU injection. Safranin-O/fast green staining of the right ankle joint for each group (**A**), bar = 200 μm. The expression levels of Sox8 (**B**, **C**), p-PI3K (**B**, **D**), PI3K (**B**, **E**), p-AKT (**B**, **F**), AKT (**B**, **G**), p-mTOR (**B**, **H**), mTOR (**B**, **I**), and Beclin1 (**B**, **J**) and the ratio of LC3-II/I (**B**, **K**) in the right ankle cartilage were detected by Western blot assay. The mRNA levels of Sox8 (**L**) in the right ankle cartilage were measured by qPCR. Results are the mean ± SD for three individual experiments. *P < 0.05, **P < 0.01.

To examine the effect of Sox8 on cartilage in vivo, gouty arthritis model rats were established by the injection of MSU crystals into the right ankle joints. Some rats were treated with lenti-Sox8 to overexpress Sox8 one day before, and sacrificed 3 days after, MSU crystal injection. Right ankle joints of the rats were stained with safranin O-fast green, which stains basophilic cartilage tissues as red by safranin-O and eosinophilic bone tissues as green or blue by fast green. The safranin-O staining in cartilage displayed the production of proteoglycan and its changes reflected the damage of cartilage. Compared with control group, the MSU crystals decreased, while overexpression of Sox8 increased, the level of proteoglycans, indicating that Sox8 alleviated cartilage damage (Fig. 8A).

### Sox8 mediated MSU crystal-induced autophagy in vivo

To verify the in vivo effect of Sox8 on PI3K/AKT/mTOR signaling pathway, the right ankle joints of rats were injected with vector or oe-Sox8 followed by the injection of 100 μL of MSU crystals (30 mg/mL) one day later. Figure 8B–L shows the expression of Sox8 (Fig. 8B, C), p-PI3K (Fig. 8B, D), PI3K (Fig. 8B, E), p-AKT (Fig. 8B, F), AKT (Fig. 8B, G), p-mTOR (Fig. 8B, H), mTOR (Fig. 8B, I), and Beclin1 (Fig. 8B, J) and the ratio of LC3-II/I (Fig. 8B, K) in the right ankle joint tissue in different groups. The mRNA level of Sox8 (Fig. 8L) was measured by real-time quantitative PCR (qPCR). The expression of Sox8 and the phosphorylation levels of PI3K, AKT, and mTOR significantly decreased in acute gout rats, whereas overexpression of Sox8 rescued the MSU crystal-induced changes. Meanwhile, the dramatically increased expressions of Beclin1 and the ratio of LC3-II/I in acute gout models were ameliorated by overexpression of Sox8. The results indicate that the in vivo effect of Sox8 on autophagy was mediated by PI3K/AKT/mTOR signaling pathway.

## Discussion

In the present study, we report that Sox8 downregulation plays an important role in MSU-induced autophagic chondrocytes via the inhibition of phosphorylation of PI3K/AKT/mTOR signaling. The findings not only underlie the mechanisms MSU induced cartilage damage, but suggest that Sox8 may serve as a potential drug in the treatment of MSU joint impairment in gout patients.

The most interesting finding is the very dramatic downregulation of Sox8 in MSU-induced chondrocytes and model rats. To our knowledge, this is the first report that Sox8 plays an important role in MSU crystal-induced cartilage damage, although SOX family members have been involved in the regulation of embryonic development and in the determination of cell fate [16]. However, the reason why the expression of SOX8 decreased after MSU treatment remains unclear. The possible mechanism may be mediated by Toll-like receptors (TLRs). It is well-established that TLR4 is present in chondrocytes [17, 18]. MSU induces intracellular response through membrane TLR2/4 and the downstream nuclear factor kappa B(NF-kB) pathway [19]. The activation of NF-kB pathway was reported to downregulate the expression of Sox9 [20]. Meanwhile, the knockdown of Sox9 reduced the expression of Sox8, indicating Sox8 is regulated by Sox9 [20, 21]. Therefore, TLRs/ NF-kB/Sox9 signaling may be the potential mechanism for MSU-induced decrease of Sox8 [20, 21].

The second important finding is that the downregulation of Sox8 induces activation of autophagy in chondrocytes by decreasing the phosphorylation levels of PI3K, AKT, and mTOR. The PI3K/AKT signaling pathway can activate mTOR by increasing the level of p-mTOR and subsequently inhibit autophagy [9, 22, 23]. When this pathway is inhibited, autophagy is activated and amplified [9, 23]. One study demonstrated that overexpression of Sox8 decreased hypoxia-induced cell injury by activating the PI3K/AKT/mTOR pathway [24]. Meanwhile, another study proved that the down-expression of Sox8 inhibited the activation of p-PI3K [16]. These two studies were in accordance with our results concerning the relationship between Sox8 and PI3K/AKT/mTOR pathway. However, how does Sox8 downregulation inhibits the phosphorylation of PI3K, AKT, and mTOR is still unrevealed. Previous studies detected that the reduction of Sox8 induced accumulation of reactive oxygen species (ROS) that suppressed the activation of PI3K/AKT pathway, making ROS a possible participator in SOX8 /PI3K/AKT/mTOR/autophagy axis [25–27].

Several limitations must be considered in the interpretation of our results. Firstly, cartilage damage in acute gout may be triggered by multiple factors. MSU crystal is a pathogenic factor, but may not be the only one. Pro-inflammatory factors including IL-1, TNF-α, and IL-6 are also involved in pathogenesis. Therefore, further studies concerning the effect of MSU crystals along with pro- inflammatory factors on cartilage are needed. Secondly, a more precisely designed in vivo study including rescue experiments may help elucidate the role of Sox8-mediated autophagy in acute gout. Thirdly, although Sox8 was originally regarded as a transcription factor, whether transcriptional activity of Sox8 contributes to MSU-induced impairment is elusive. According to our result, the downregulation of Sox8 decreased the phosphorylation of PI3K, AKT, and mTOR, while the total expression of PI3K, AKT, and mTOR was not changed. The phosphorylation of proteins usually spreads fast, whereas transcription is usually a slow process, implying an enzymatic but not transcriptional effect of Sox8 [28]. To identify the specific role of Sox8, further studies to detect the dynamic expressions of PI3K/AKT/mTOR/autophagy axis protein at different time points in Sox8 overexpressed cells may be needed.

Collectively, the present study indicates that Sox8 downregulation inhibits the PI3K/AKT/mTOR signaling pathway and increases the level of autophagy in MSU-induced impairment of chondrocytes in vitro and in vivo, making Sox8 a potential treatment target for gout-induced osteoarthritis.

## Materials and methods

### Reagents and materials

MSU crystal was purchased from Sigma (*U2875*-5G, St. Louis, MO, USA). Modified safranin O-Fast Green FCF Cartilage Stain Kit was obtained from Solarbio (G1371, Beijing, China). CCK-8 Cell Proliferation and Cytotoxicity Assay Kit were purchased from Beyotime (C0037, ShangHai, China). DAPI staining solution was from KeyGEN Biotech (KGA215, NanJing, China). Anti-Beclin1 antibody (ab207612), anti-β-actin antibody (ab8226), anti-Sox8 antibody (ab104245), goat anti-rabbit IgG H&L (HRP; ab6721), and rabbit anti-mouse IgG H&L (HRP; ab6728) were purchased from Abcam (Cambridge, the UK). LC3A/B (D3U4C) XP® rabbit mAb (12741), PI3K antibody (4257), AKT antibody (9272), p-AKT antibody (9271), p-mTOR (Ser2448) antibody (2971), and mTOR antibody (2972) were obtained from Cell Signaling Technology (Boston, MA, USA). p-PI3K antibody (AF3241) was from Affinity Biosciences (OH, USA). Monodansylcadaverine (MDC, KGATG002) was purchased from KeyGEN Biotech (NanJing, China). Hematoxylin eosin staining kit (KGA224) was obtained from KeyGEN Biotech (NanJing, China). Lipofectamine RNAiMAX transfection reagent (13778100) were purchased from Thermo Fisher Scientific(Pittsburgh, PA, USA).siSox8 plasmid (sc-38418) and siRNA-NC Plasmid (sc-37007) were purchased from Santa Cruz (CA, USA).

### Cell culture and MSU crystal preparation

Human chondrocyte cell line (C28/I2 cell) was purchased from OTWO BIOTECH Company (Shenzhen, China). The cell line used was tested for mycoplasma contamination and authenticated by STR profiling.

C28/I2 cells were cultured in DMEM medium containing penicillin (final concentration of 100 U/ mL), streptomycin (final concentration of 100 μg/ mL), and 10% fetal bovine serum. The cells were incubated with MSU crystals (200 μg/ml) for 24 h as the in vitro model.

For in vitro study, 20 mg MSU powder was dissolved in 1 mL Tween 80 with 9 ml DMEM as a stock solution that was autoclaved and stored at 4 °C.

### Cell transfection

The Plvx-puro plasmid vector was obtained from Addgene Co. (Watertown, MA, USA). The PHelper 1.0 and PHelper 2.0 plasmid, and transfection reagent were purchased from GENE (ShangHai, China). C28/I2 cells (0.5 × 10^5^ cells per well) were transfected with control lentivirus and lenti-Sox8 for 48 h. The transfection efficiency of Sox8 overexpression in C28/I2 cells is shown in Supplementary Fig. 2.

Plasmids were transfected with Lipofectamine RNAiMAX transfection reagent. Transfection was performed using sterilized mother liquor with a concentration of 20 μM.5 μl siRNA-NC or siSox8 for 48 h. After transfection, PCR assay was performed to detect the transfection efficiency (Supplementary Fig. 3).

### Animal model, overexpression of Sox8, and RNA sequencing

Sprague Dawley rats (160–200 g) were purchased from Changzhou Cavens Laboratory Animal Co., Ltd. (http://www.cavens.com.cn/; Changzhou, Jiangsu, China). All rats were bred under set conditions (22 °C ± 2 °C and light/dark cycle at 12 h intervals) and given free access to water and food. The investigator was blinded to the group allocation during the experiment and/or when assessing the outcome.

The rats were randomly assigned into four groups (*n* = 3) after a 7-day acclimatization. In the model of acute gout arthritis, 100 μL of MSU crystals (30 mg/mL) was injected into the right ankle joint on the first day. The rats in the control group received 100 µL of saline. Some rats were injected with lenti-Sox8 on day 0 to overexpress Sox8.

For RNA sequencing of articular cartilage, samples were procured on the third day and sequenced with an Illumina Novaseq 6000.

### Cell viability assay

Cell viability was assessed by the CCK-8 assay. In brief, cells of indicated groups were seeded into 96-well culture plates and incubated for 24 h. Then, 100 mL of 10% CCK-8 solution was added to each well and incubated for another 2 h at 37 °C. Formazan absorbance was assessed by an auto microplate reader (Berthold LB941) at a wavelength of 450 nm.

### Autophagy detection

Autophagic flux in C28/I2 cells was detected using the mRFP-GFP-LC3 adenovirus (Hanbio Co. Ltd., Shanghai, China). C28/I2 were plated in a 24-well plate at a density of 1 × 10^6^ cells/well and incubated with 50 mL of mRFP-GFP-LC3 adenovirus overnight. The cells were then treated with 0.3% Triton X-100 for 10 min. Autophagic flux was observed under a Zeiss LSM confocal laser microscope (Zeiss, Germany). The yellow puncta indicated autophagosomes, and the red puncta indicated autolysosomes.

MDC staining was applied to evaluate autophagic vacuoles. 100 µL of MDC staining solution was added to each well. After being kept at room temperature for 15–45 min in the dark, the cells were washed and suspended, followed by flow cytometry (Beckman DxFlex, USA).

The morphology of autophagosomes in C28/I2 cells was observed by TEM. Cells were fixed with a solution containing 2.5% glutaraldehyde for 1 h. The sections were first stained with lead citrate for 10 min and then with uranyl acetate for 30 min. Zeiss TEM was used for the observation.

### qPCR

The mRNA expression was quantified by qPCR. Following the instructions of the manufacturer, total RNA was extracted from C28/I2 cells or cartilage by TRIzol reagent. qPCR was performed using the 20 μL system, which included cDNA (1 μL), nuclease-free water (7.4 μL), Ssofast EvaGreen Supermix (10 μL), forward primer (0.8 μL), and reverse primer (0.8 μL). Fold change (2−ΔΔCt) was used to analyze the relative expression of target genes [29] and GAPDH expression as the inner standard to normalize the expression. Each gene analysis was performed in triplicates. The qPCR primers are shown in Table 1.

**Table 1:** Primers used in quantitative real-time qPCR.

Primers	Sequence (5’→3’)	
SOX8 (Human)	Forward	TGTACAAGGCTGAAGCAGGG
	Reverse	CTGAGCTCCGAGATGTCCAC
Sox8 (Rat)	Forward	CATGTACAAAACGGACACAG
	Reverse	TGTTACTGATAACCTCGCTG
GAPDH (Human)	Forward	AATGAATGGGCAGCCGTTA
	Reverse	TGTAAACCATGTAGTTGAGGT
GAPDH (Rat)	Forward	CTGCTCCTCCCTGTTCTA
	Reverse	GTGGGTAGAGTCATACTGGA

### Western blot assay

Total protein from C28/I2 cells and cartilage was extracted with RIPA lysis buffer. A bicinchoninic acid protein assay kit was used to quantify total protein. Samples were separated by SDS-PAGE and transferred onto a polyvinylidene difluoride (PVDF; nitrocellulose) membrane in Tris-glycine buffer. After removing the bubble, the membrane was dyed with Ponceau-S for 5 min and then cleaned twice with Tris-buffered saline/Tween 20 (TBST). The membranes were blocked with 5% fat-free milk-blocking buffer for 2 h at room temperature and then incubated overnight at 4 °C with respective primary antibodies. After washing with TBST, the membranes were incubated with HRP-labeled secondary antibodies. The membranes were exposed to equal volumes of mixed chemiluminescent reagents A and B for 5 min. Tanon 6600 luminescent imaging workstation was used for detection. The experiments were repeated at least three times.

### Immunohistochemistry

Tissues were fixed in formalin paraffin-embedded and immunohistochemically stained with primary anti-Sox8 antibody (1:1000, bs-11600R, Bioss Antibodies, Beijing, China) as described [30, 31]. The slides were evaluated using the Olympus inverted phase contrast microscope. Safranin-O-fast green (Solarbio, Beijing, China) was used to stain the cartilage matrix and the specimens were examined using a phase contrast microscope (Olympus Optical, Japan).

### Quantification and statistical analyses

All experiments were performed independently and repeated at least three times. The results were expressed as means ± SD. GraphPad Prism software (version 7.0, La Jolla, CA, USA) was used to perform all analyses. The statistical significance was assessed by one-way ANOVA and Tukey’s test among multiple groups. A value of *P* < 0.05 was considered statistically significant. (*P < 0.05, **P < 0.01, and ***P < 0.001).

## Supplementary information

Original Data File

supplementary material legends

Supplementary Figure 1

Supplementary Figure 2

Supplementary Figure 3

## Data Availability

The original data generated and/or analyzed during the current study are available from the corresponding author upon reasonable request.
