# Supplementary material for: Downregulation of Sox8 mediates monosodium urate crystal-induced autophagic impairment of cartilage in gout arthritis
Source: Cell Death Discov. 2023 Mar 14;9:95. doi: 10.1038/s41420-023-01388-z (PMC10015026; doi:10.1038/s41420-023-01388-z)

Fig3

Beclin1

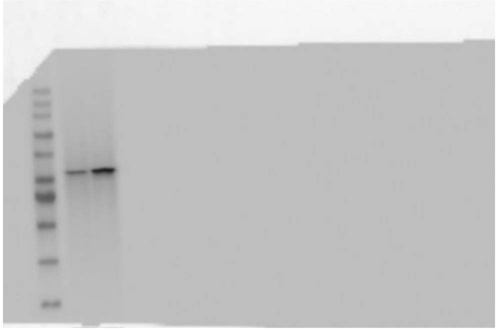

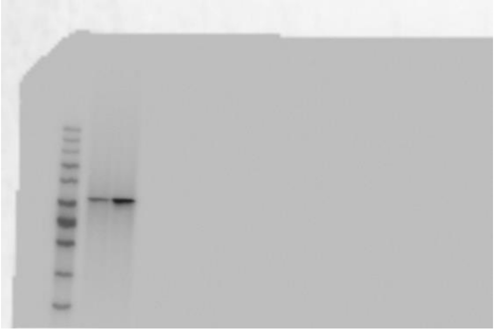

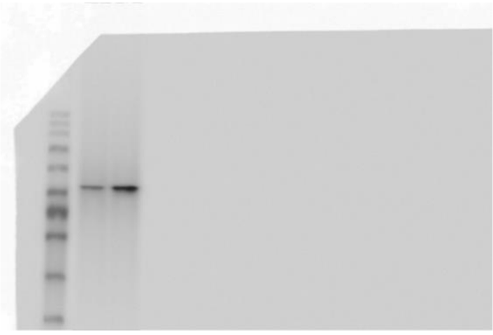

βactin

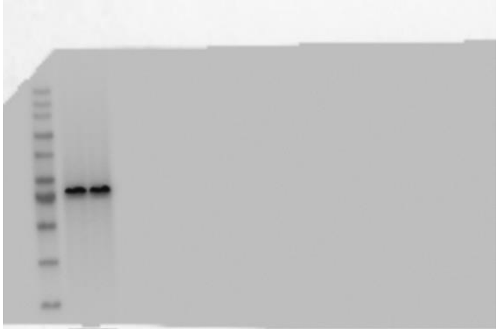

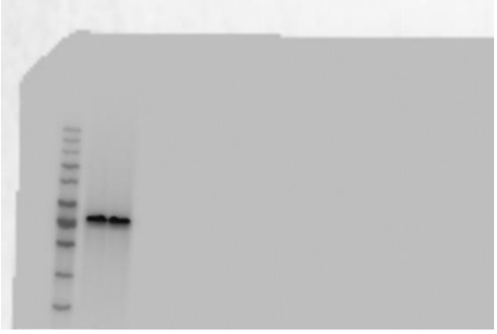

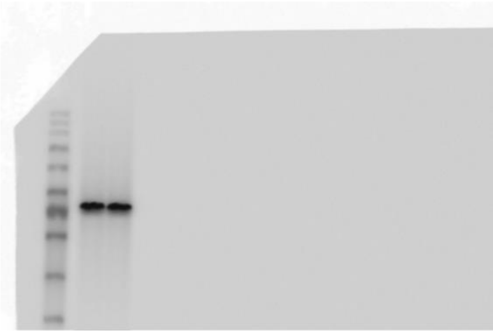

LC3II/I

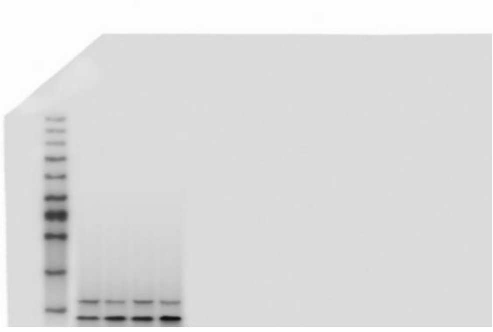

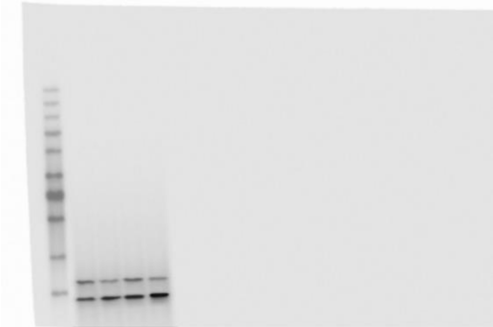

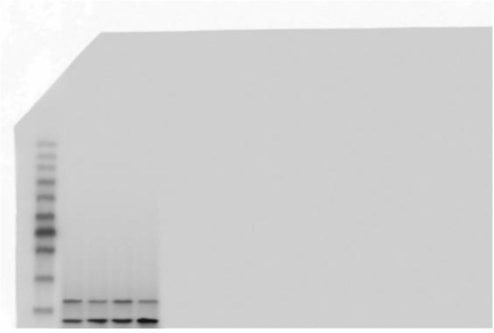

βactin

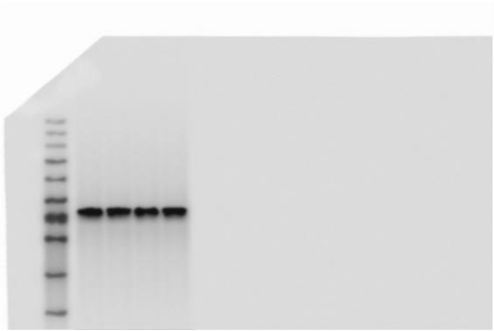

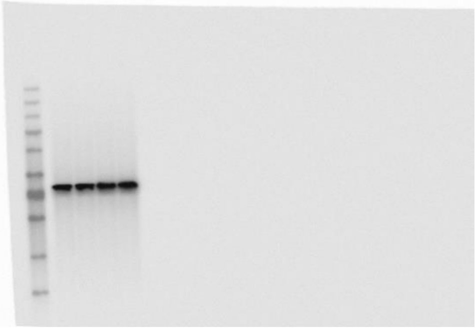

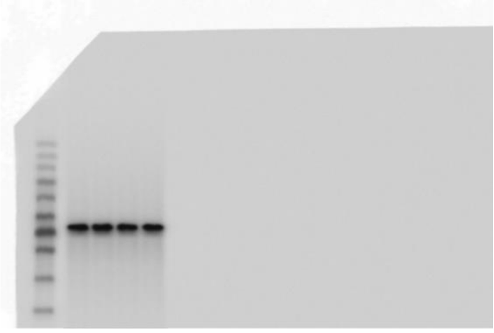

FIG4

AKT

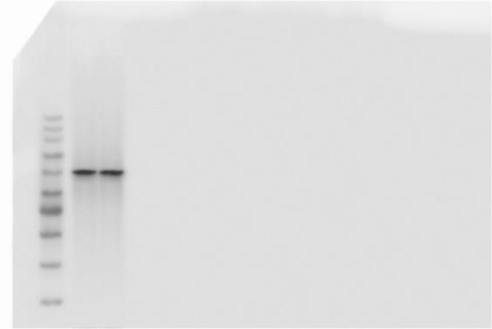

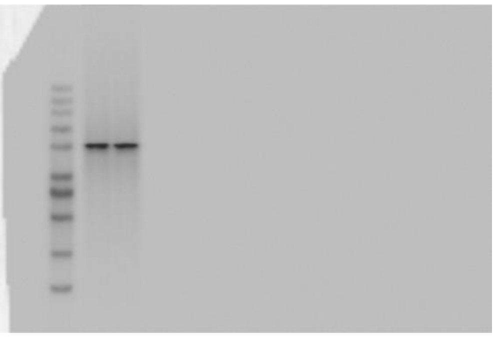

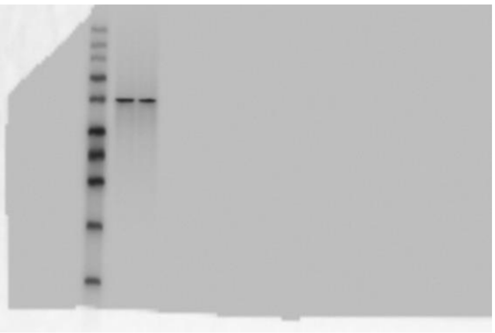

MTOR

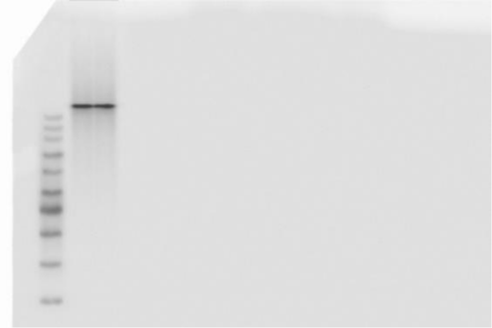

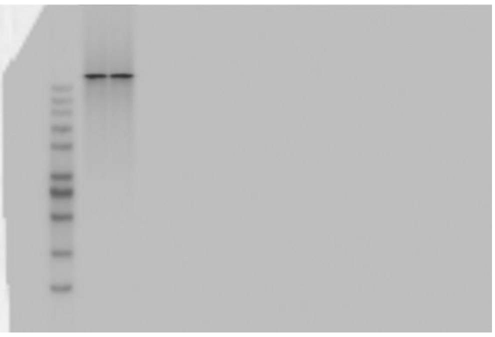

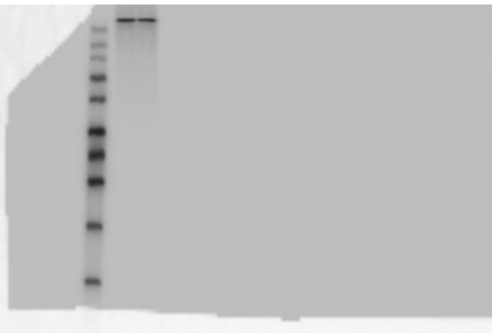

p-AKT

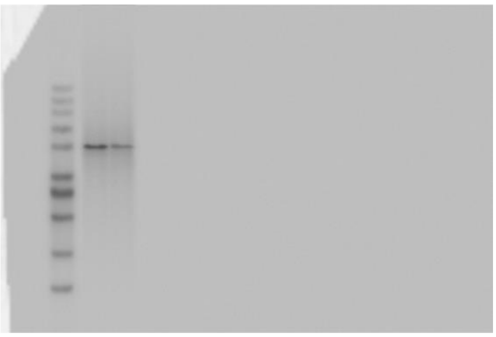

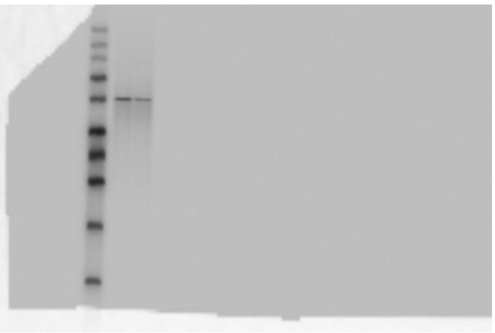

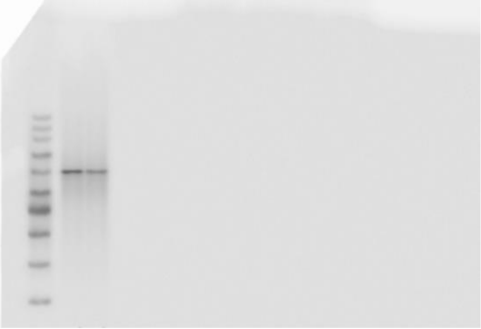

pmTOR

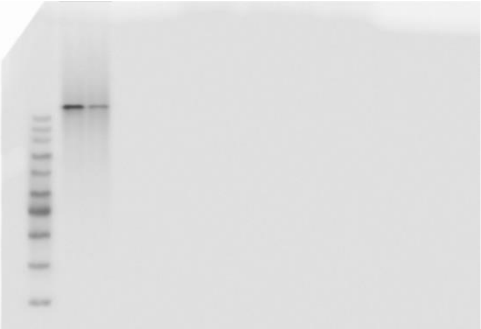

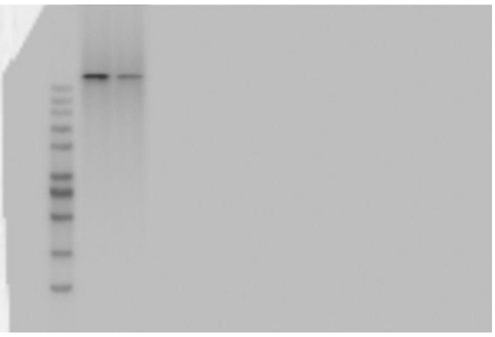

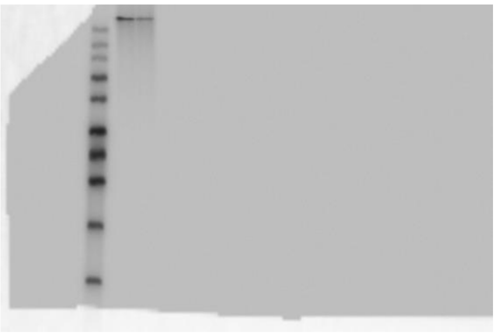

PI3K

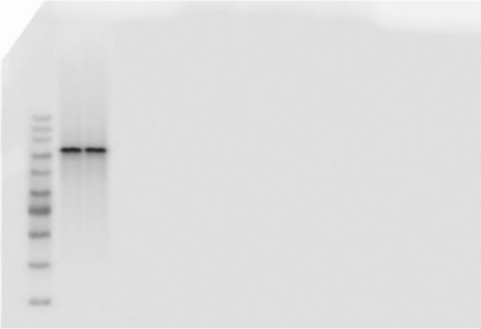

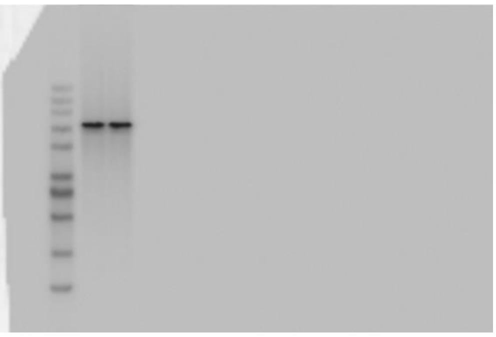

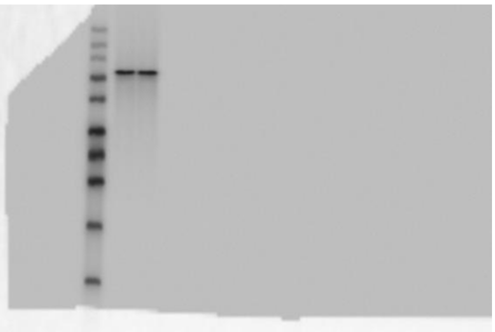

p-PI3K

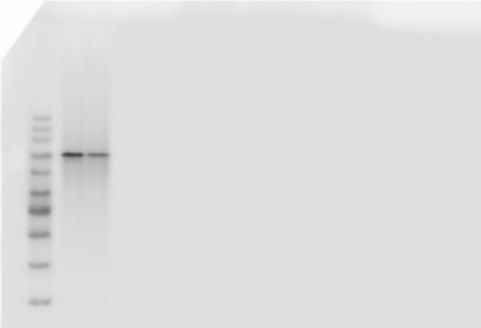

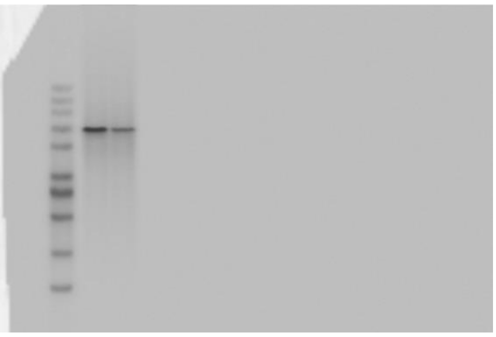

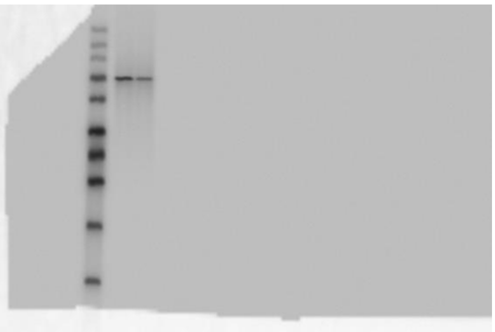

Sox8

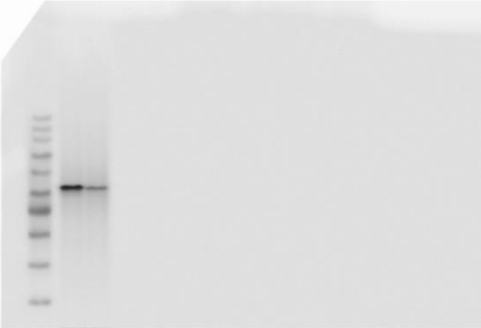

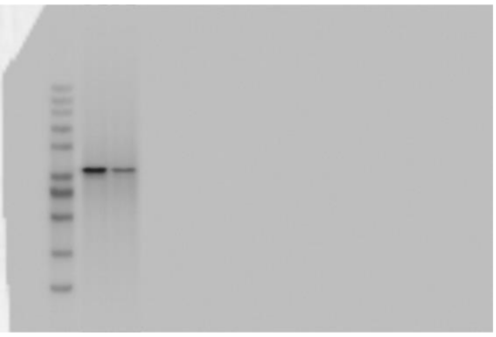

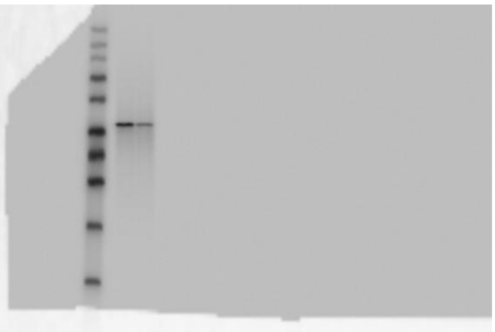

βactin

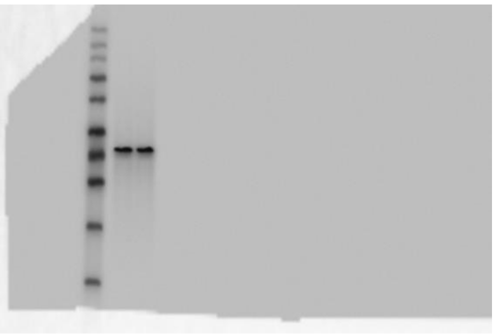

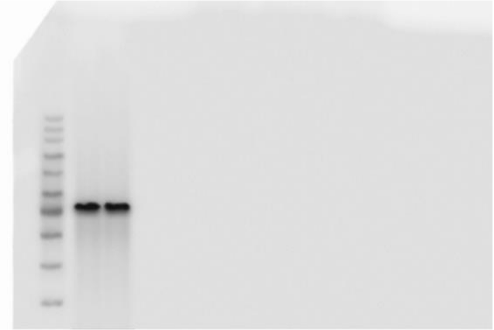

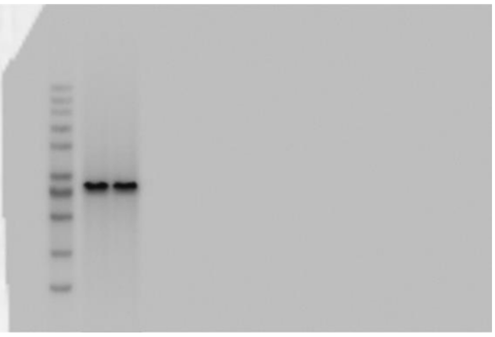

Fig6

OE-Sox8

Beclin1

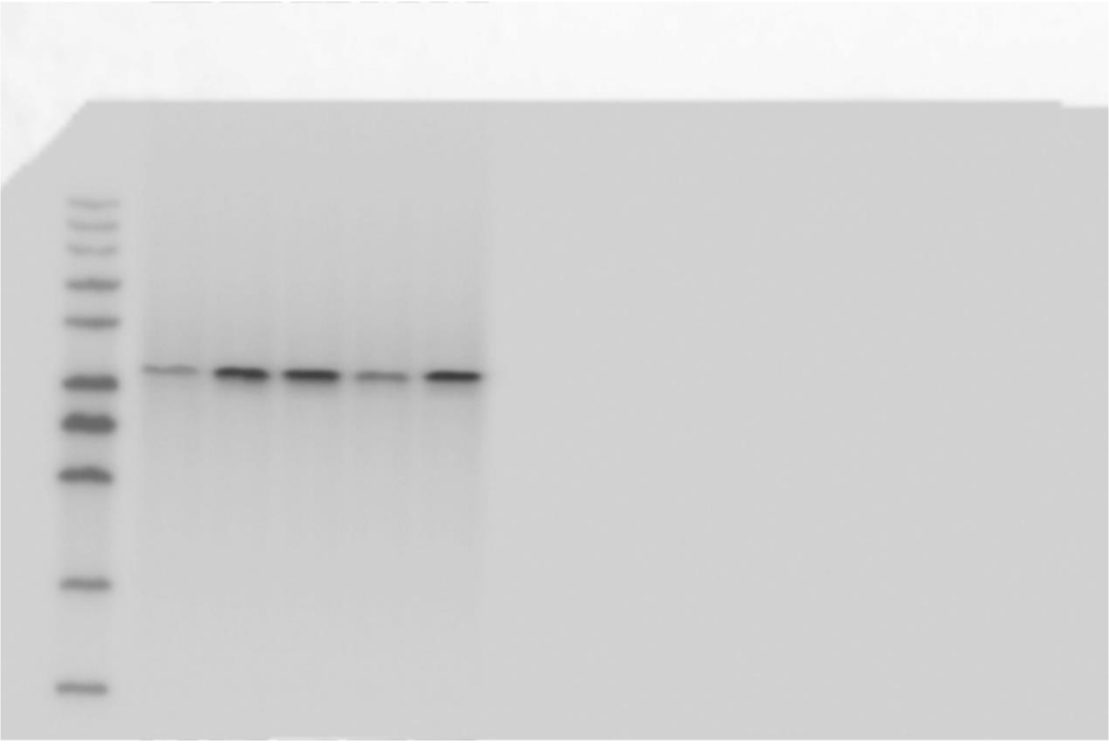

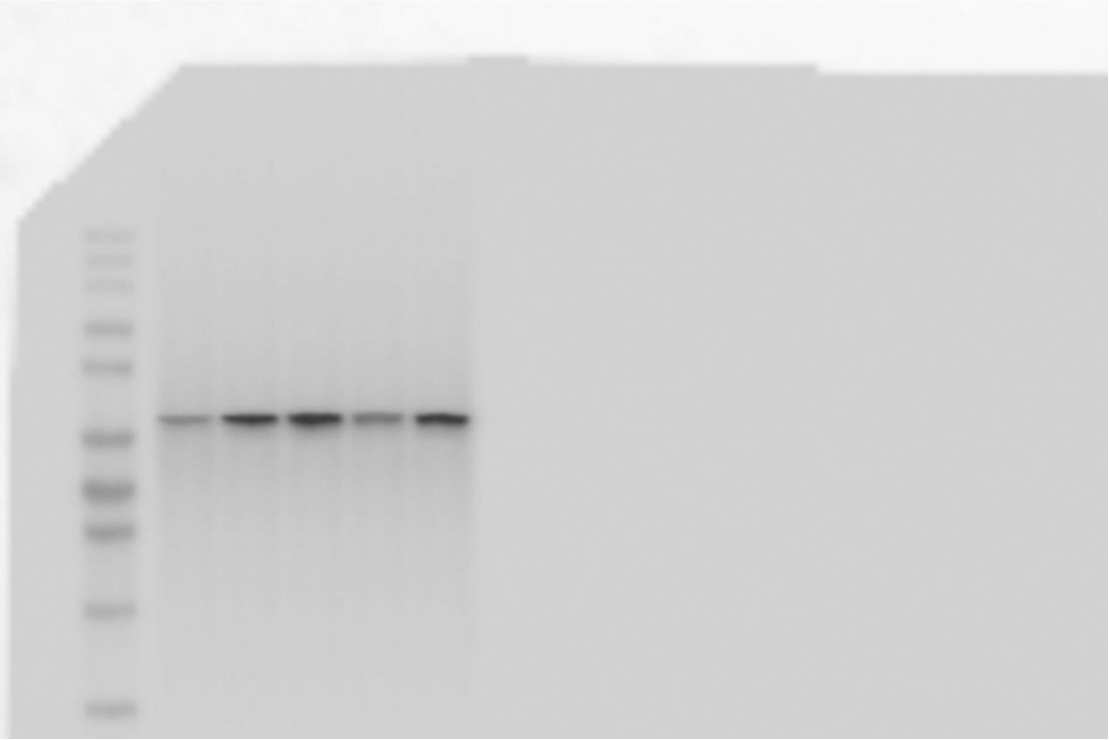

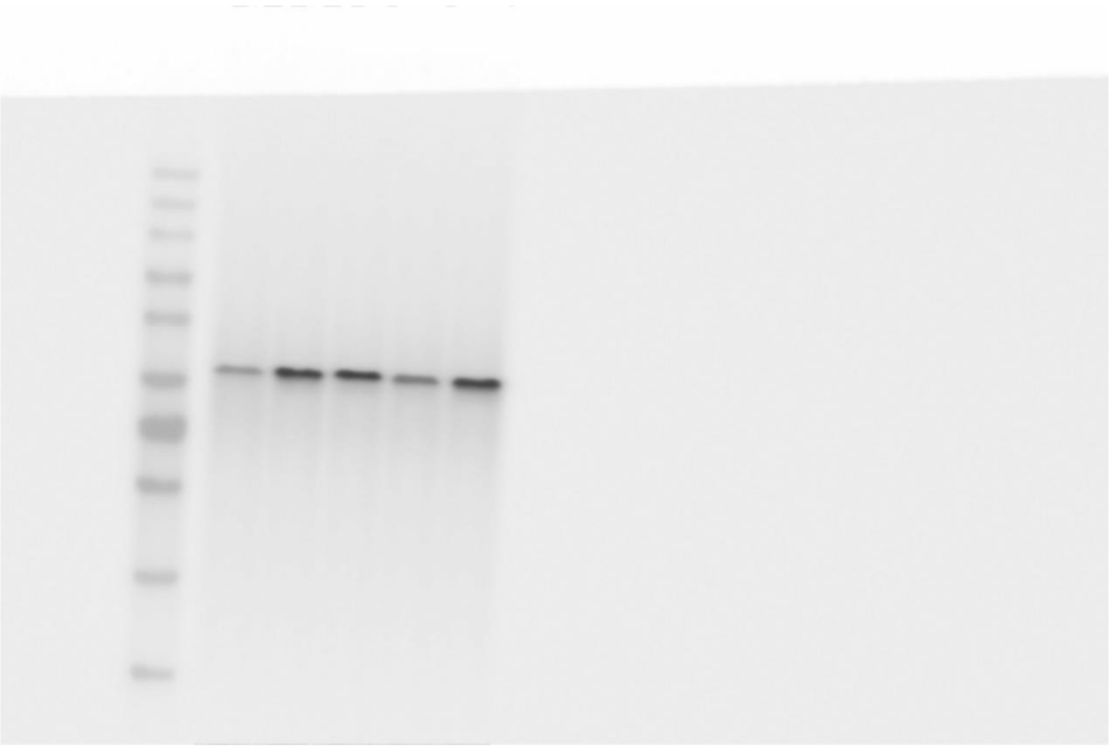

βactin

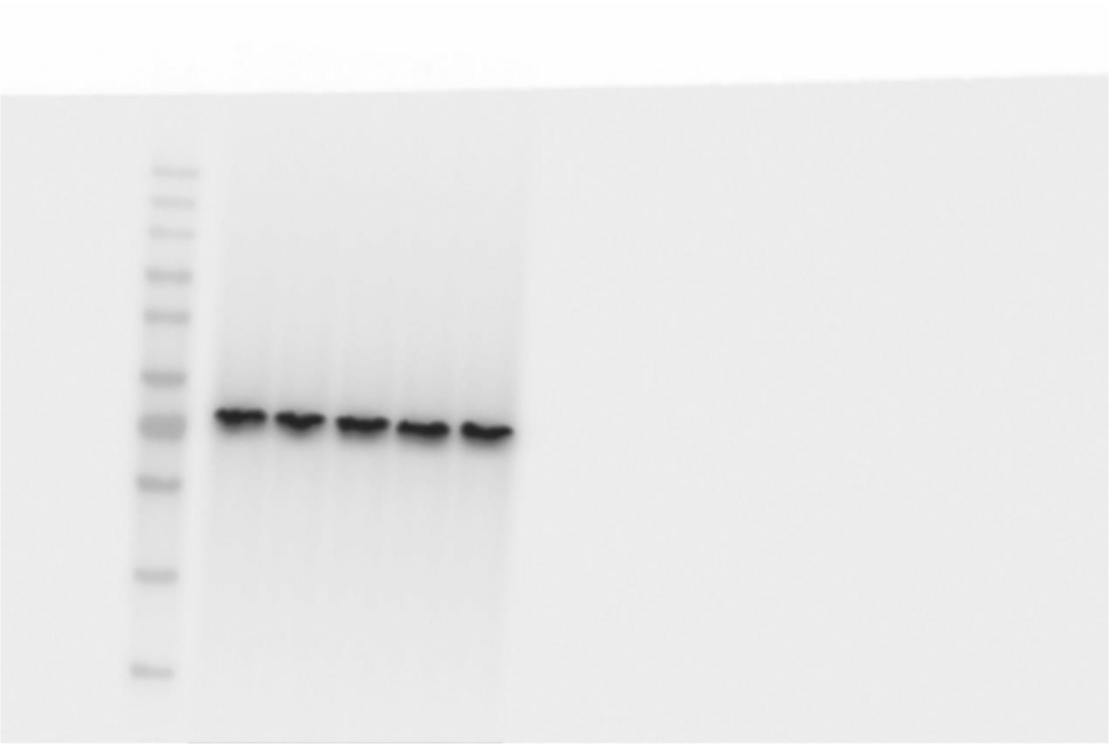

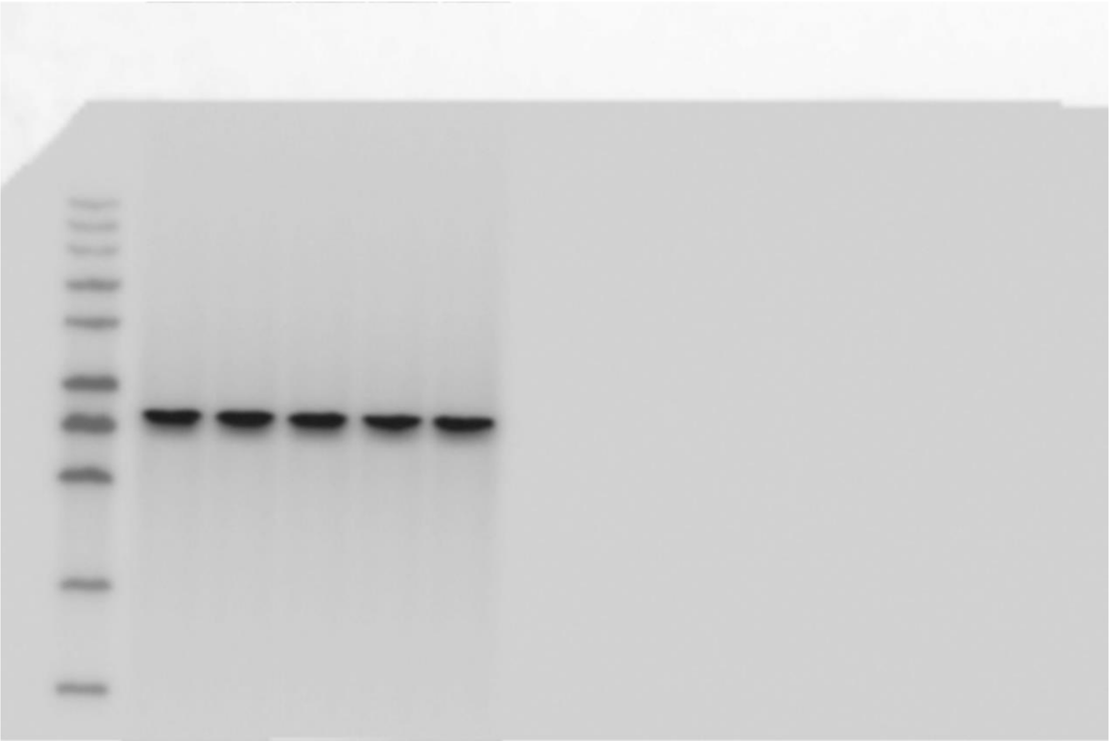
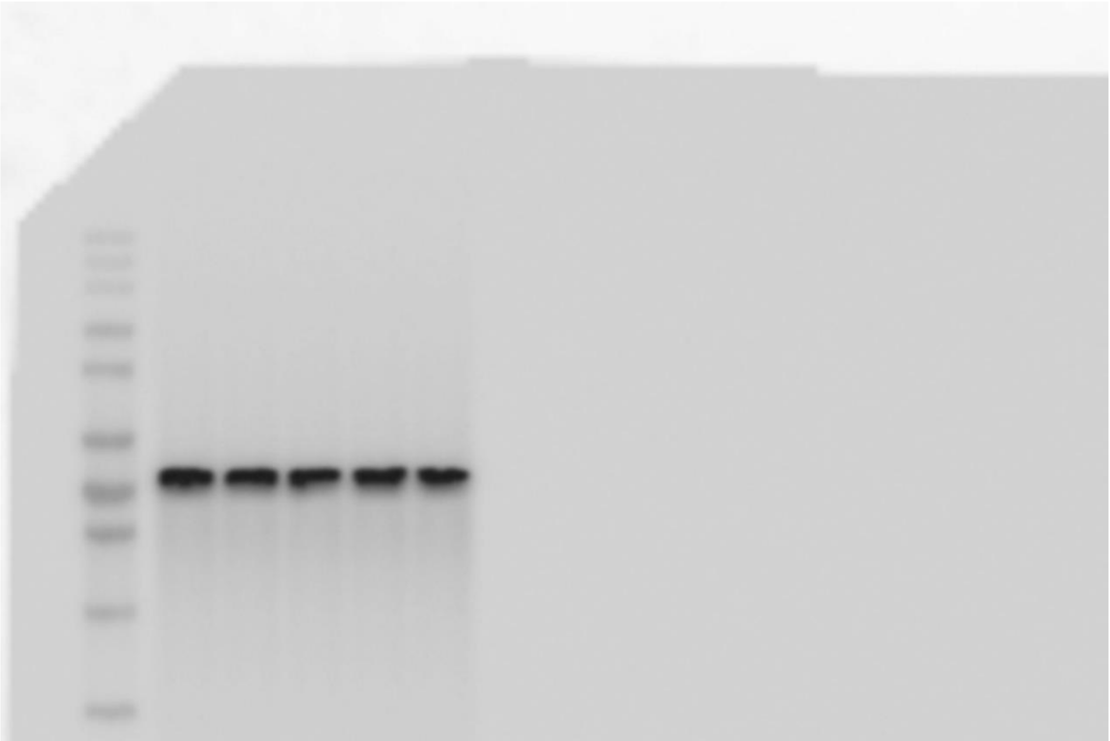

OE-Sox8

LC3II/I

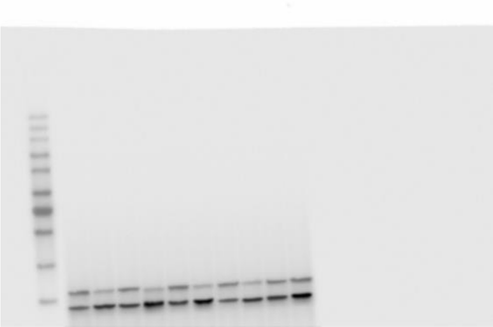

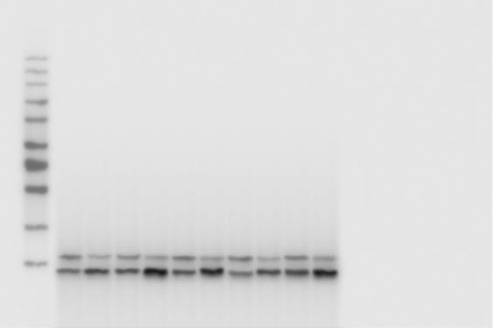

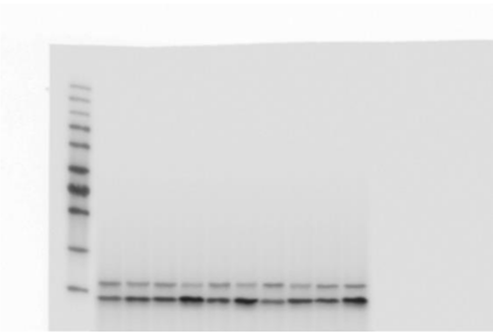

βactin

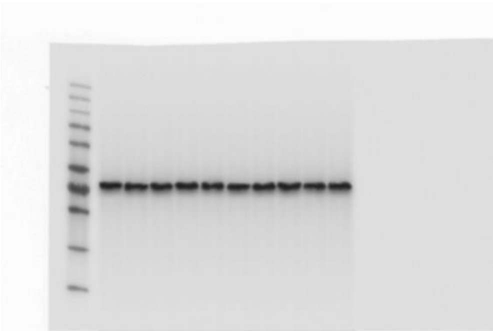

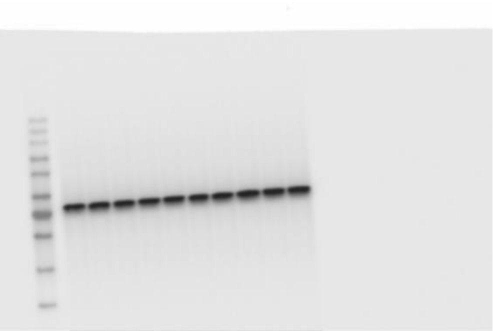

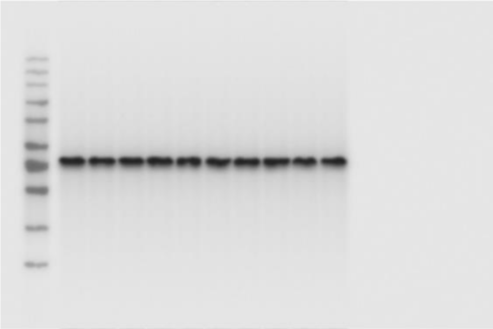

siSox8

ATG5

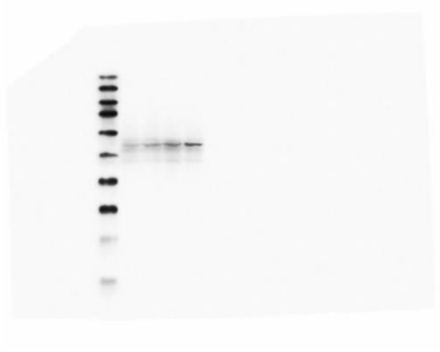

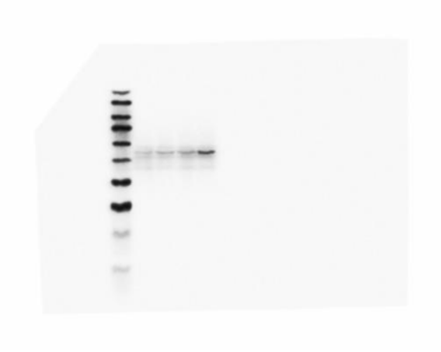

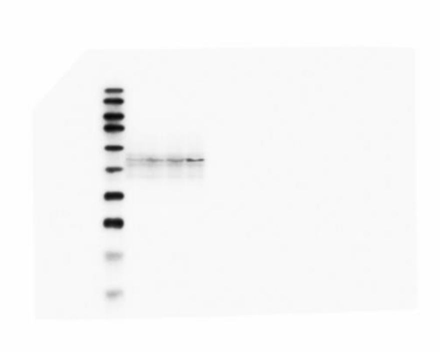

Beclin1

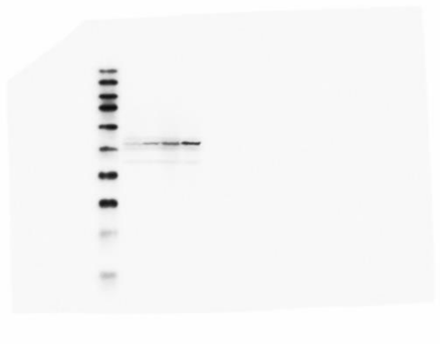

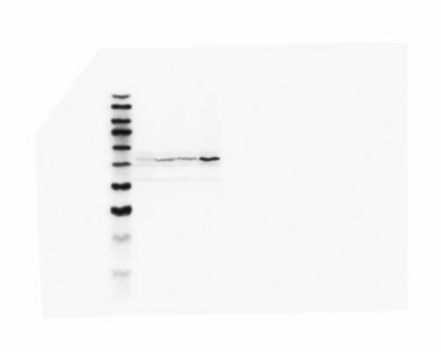

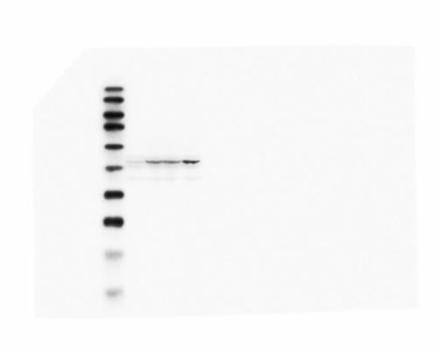

βactin

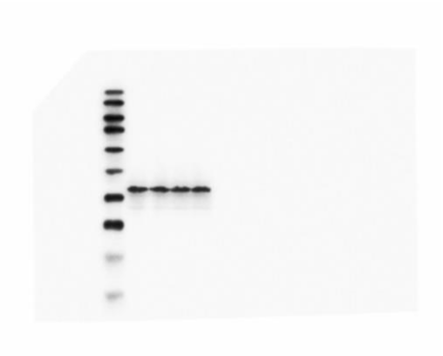

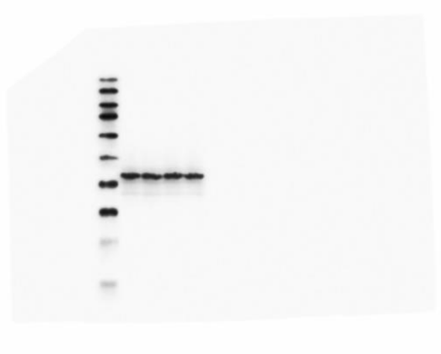

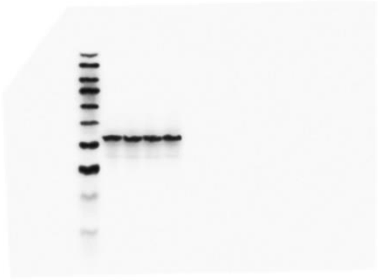

siSox8

LC3II/I

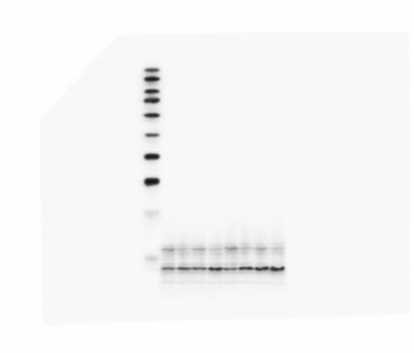

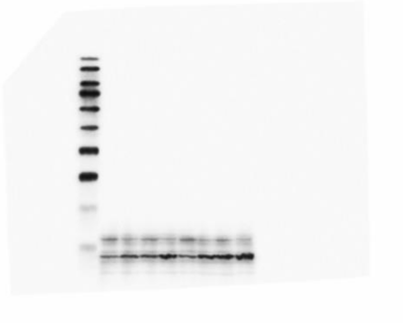

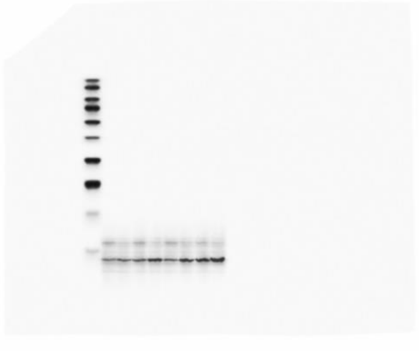

βactin

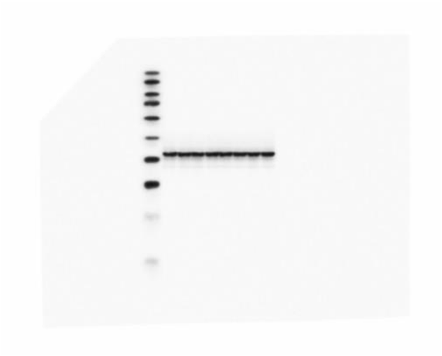

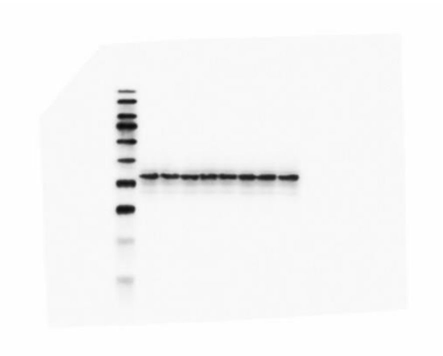

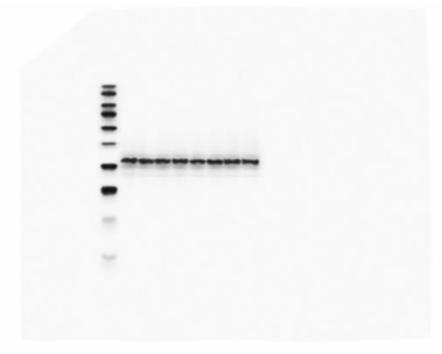

FIG7

OESox8

AKT

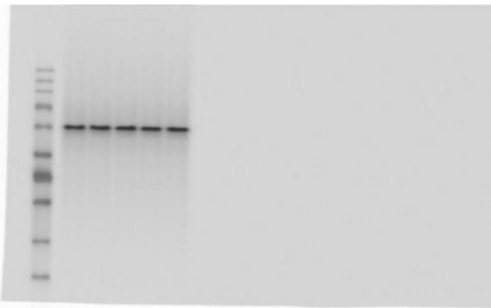

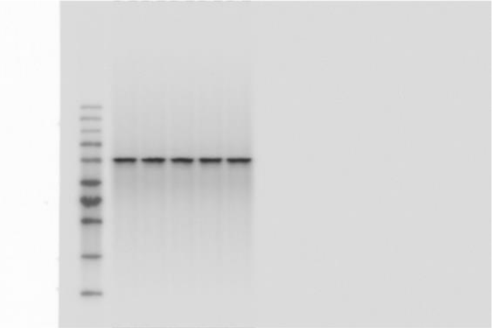

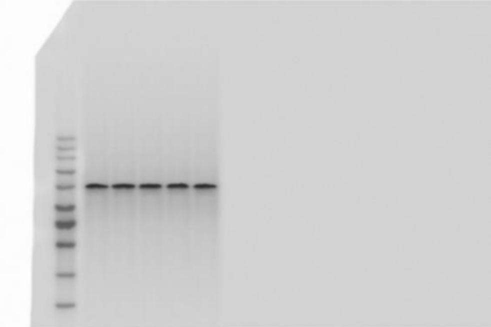

mTOR

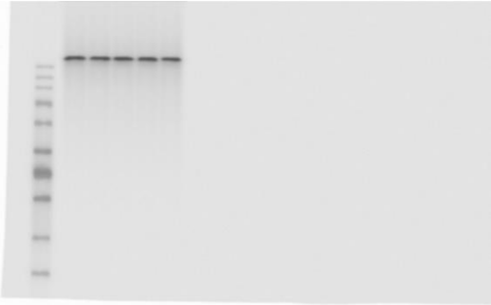

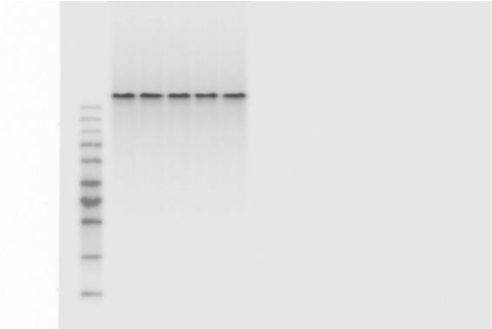

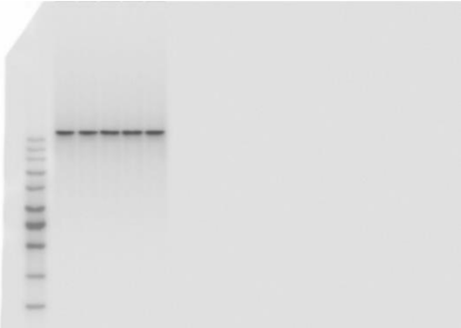

p-AKT

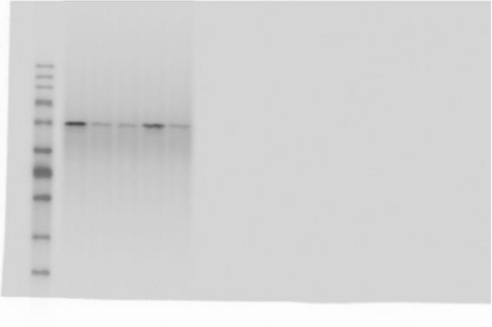

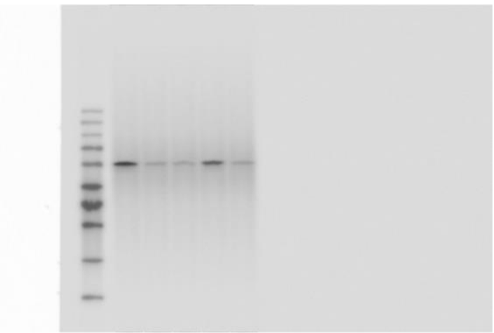

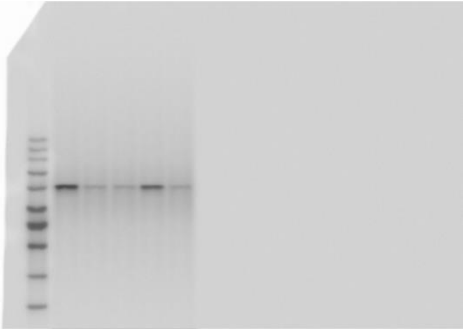

p-mTOR

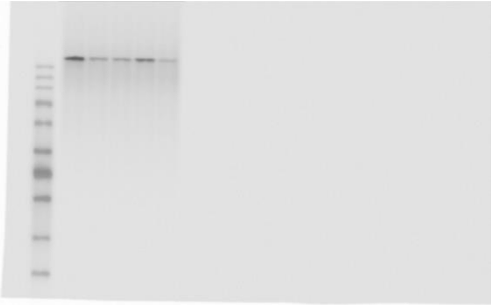

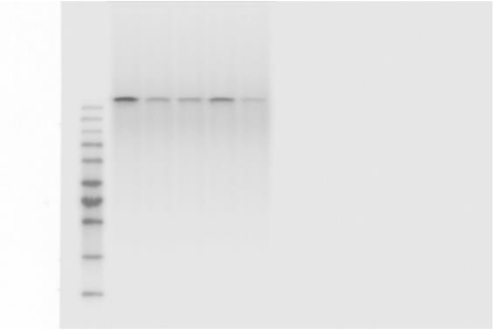

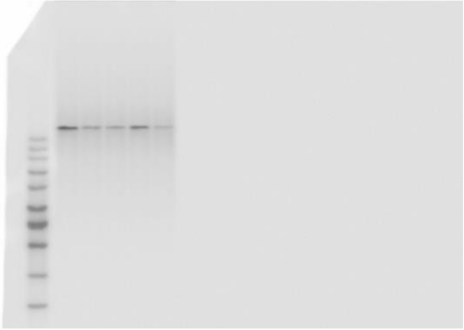

p-PI3K

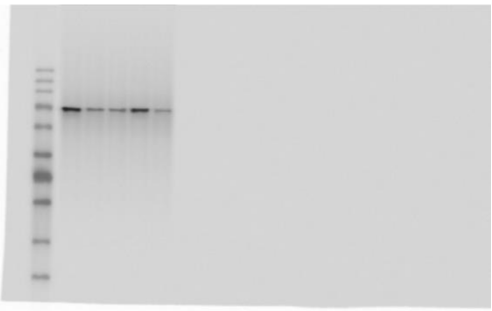

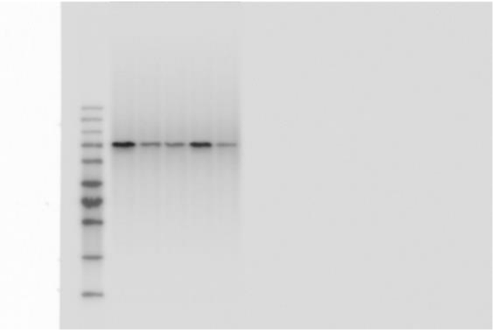

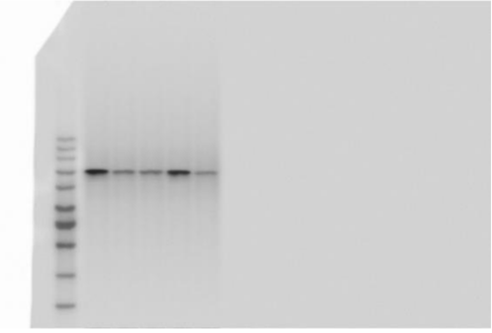

βactin

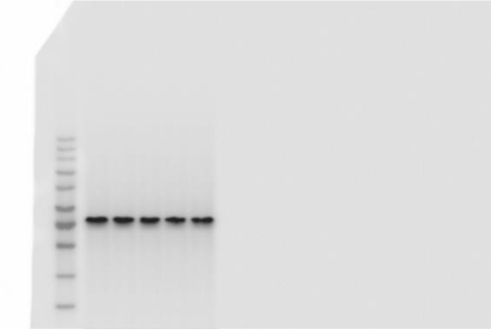

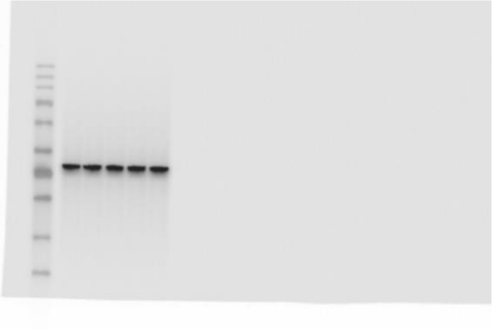

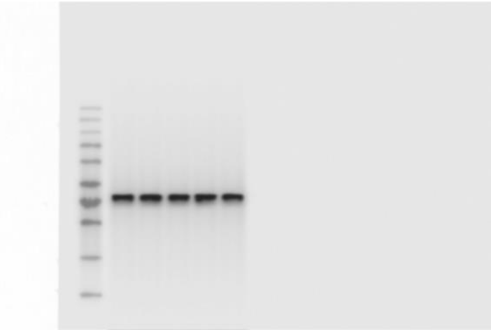

siSox8

AKT

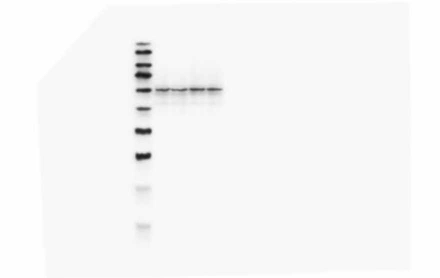

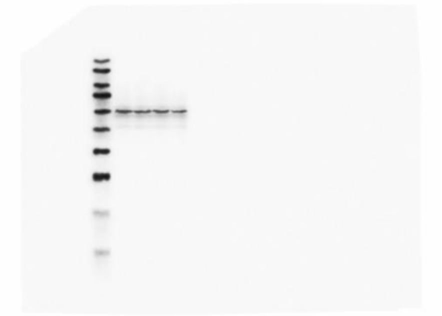

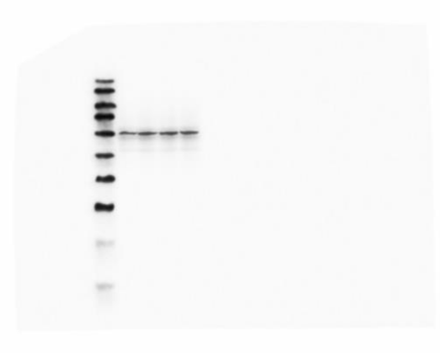

mTOR

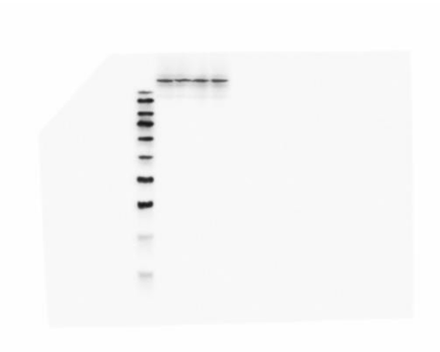

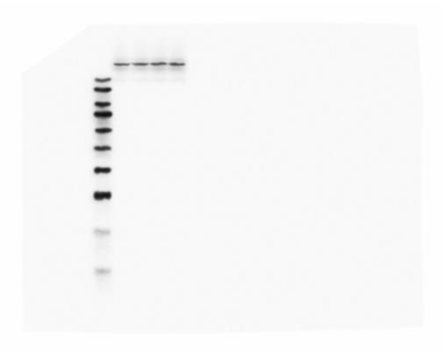

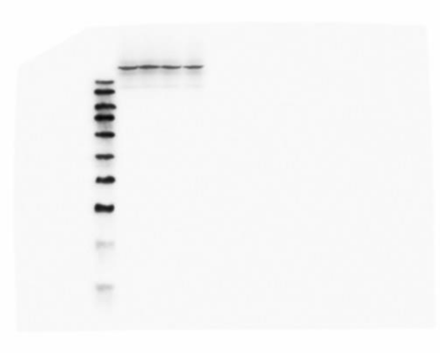

p-AKT

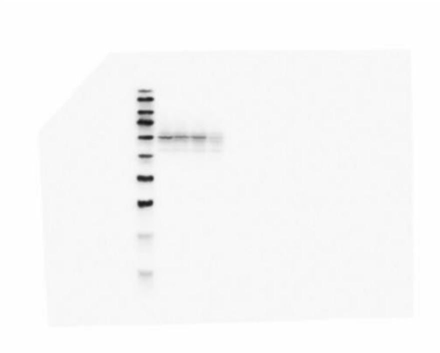

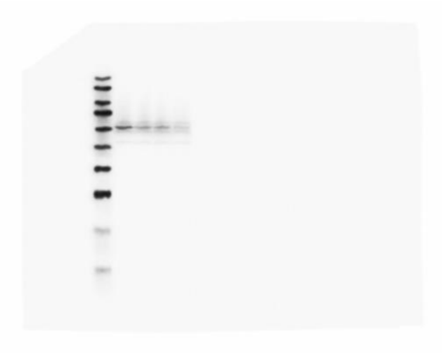

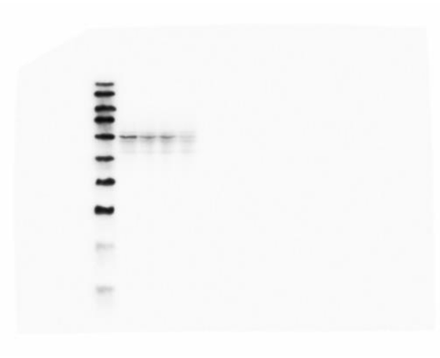

PI3K

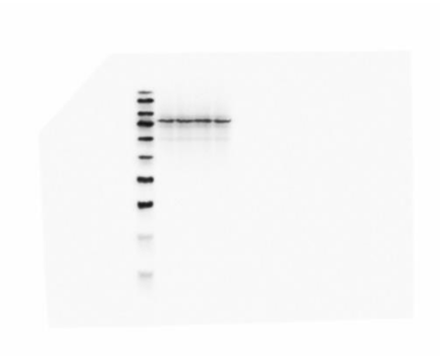

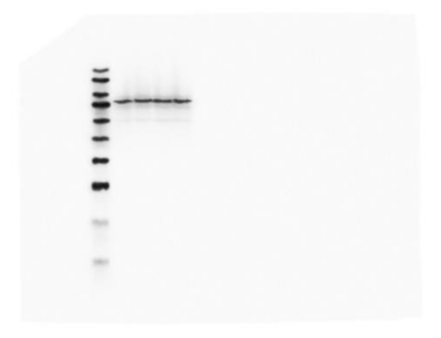

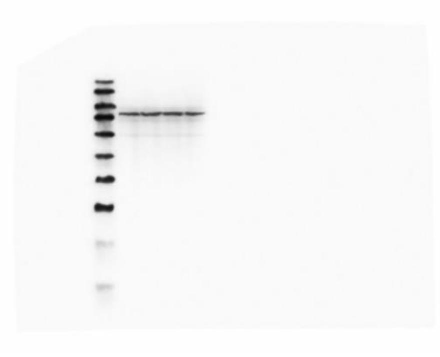

p-mTOR

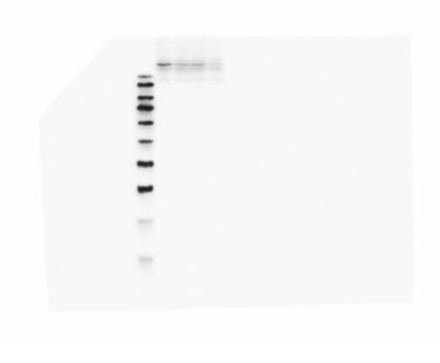

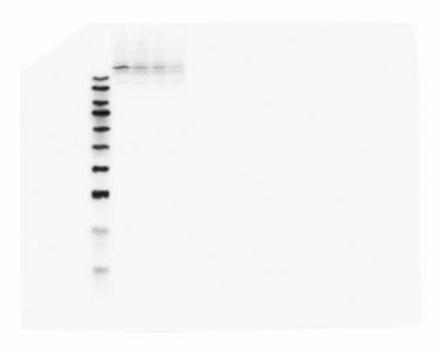

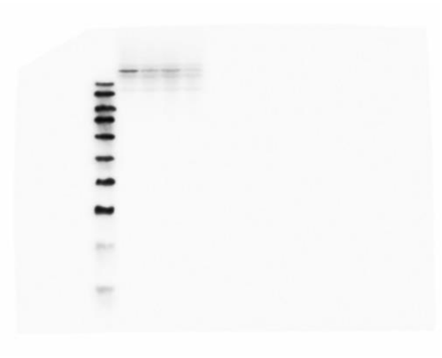

p-PI3K

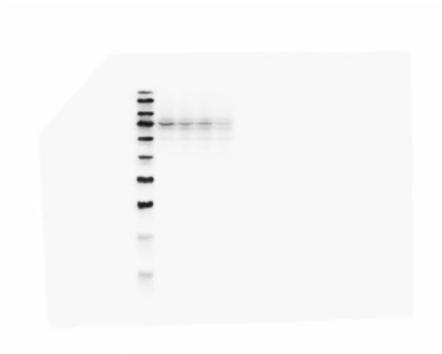

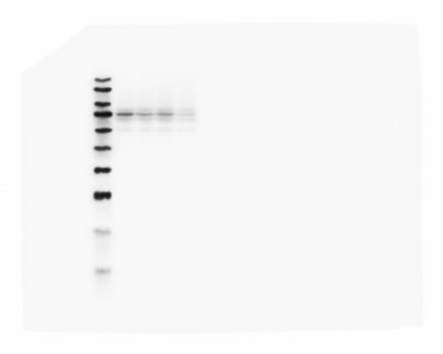

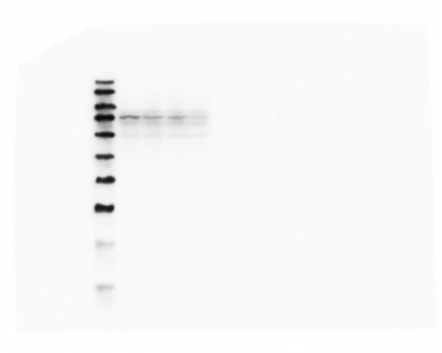

βactin

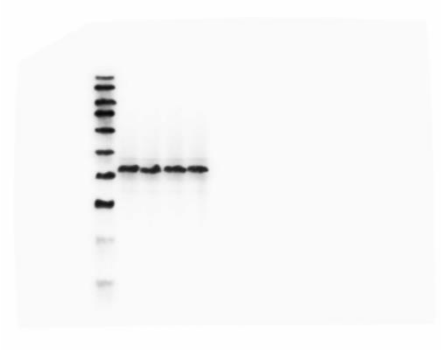

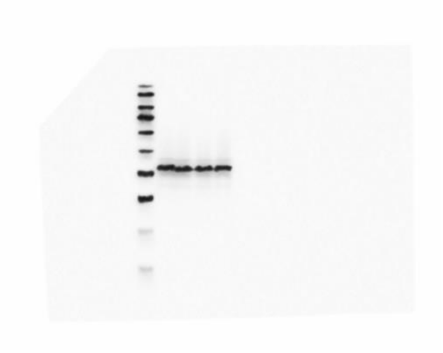

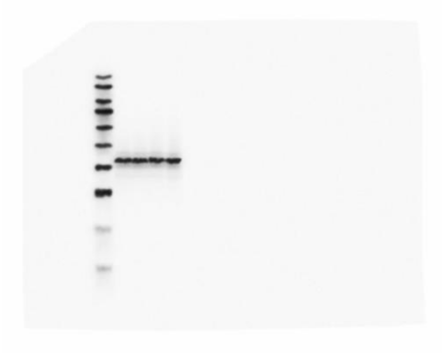

Fig8

AKT

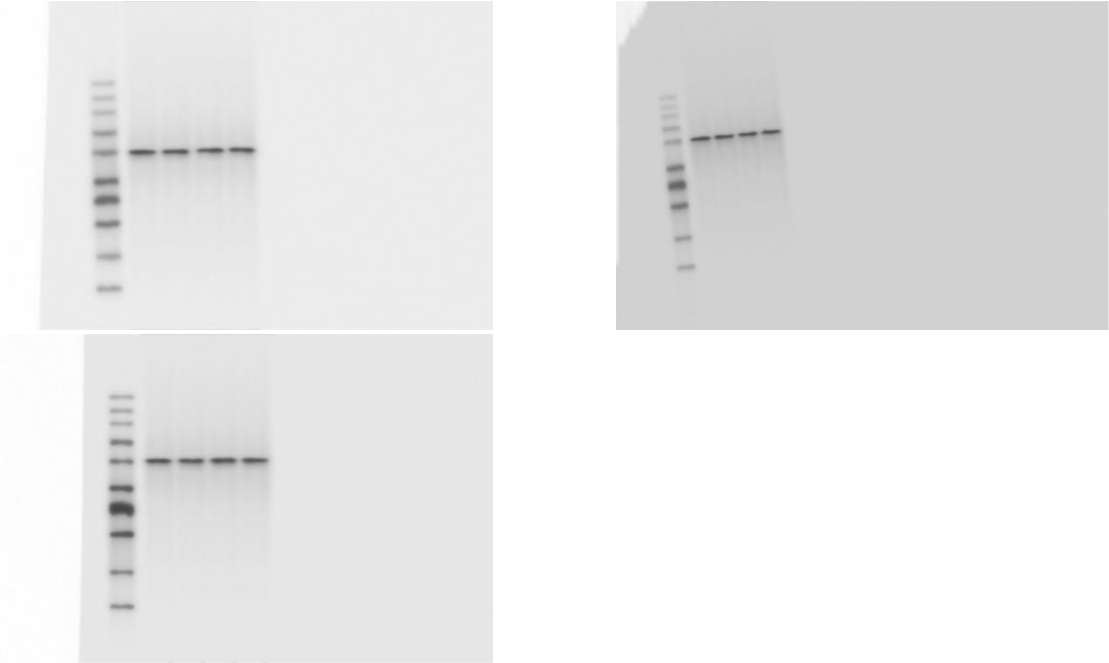

p-AKT

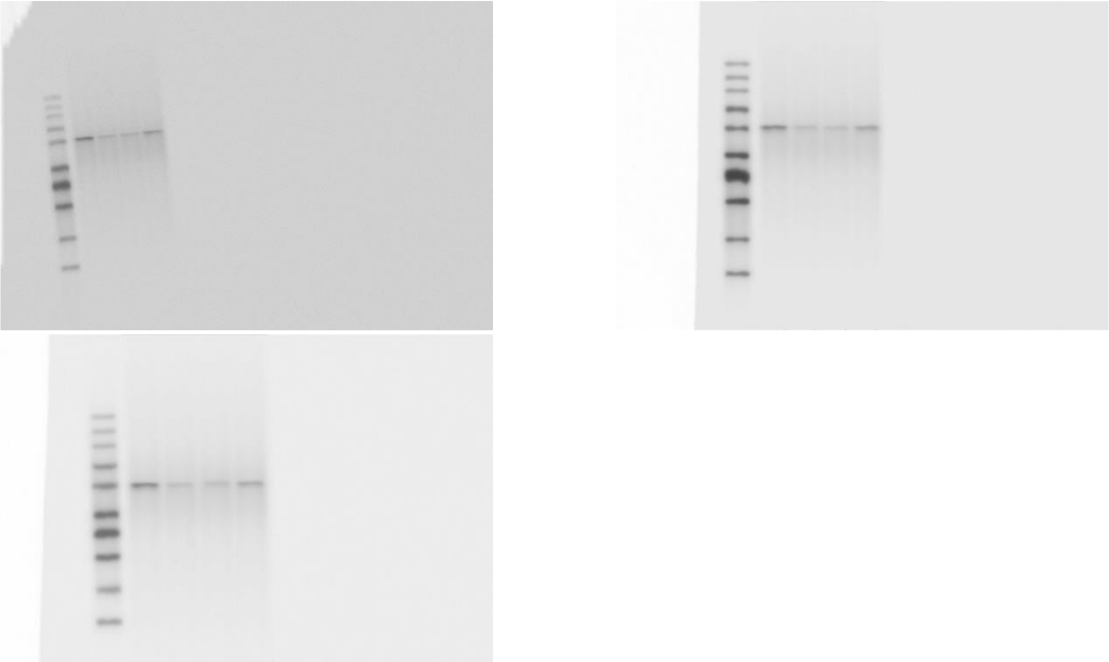

PI3K

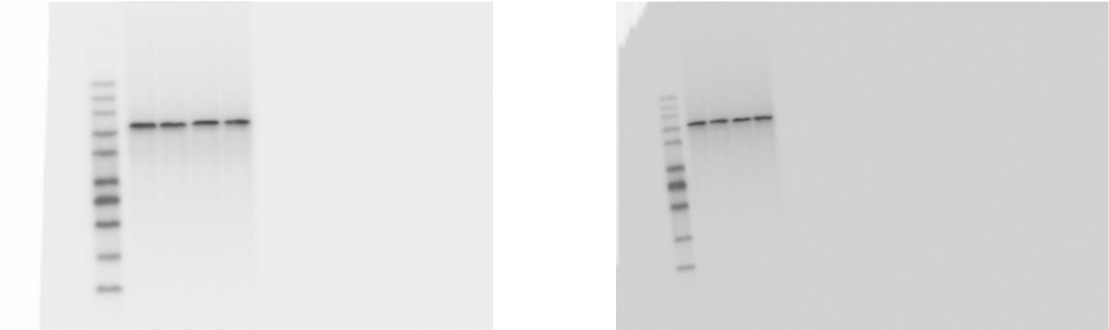

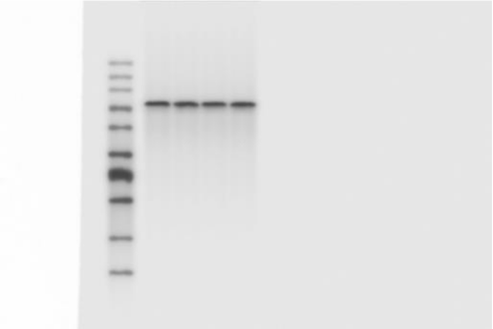

p-PI3K

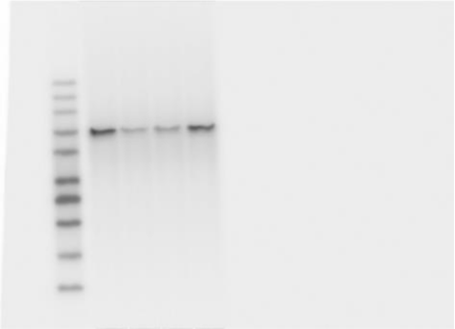

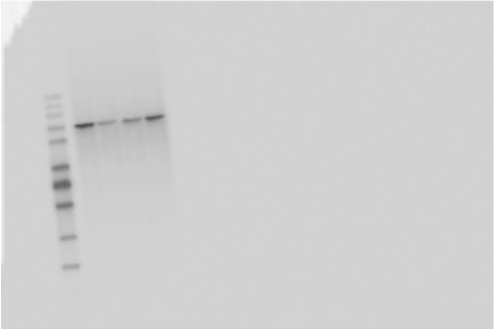

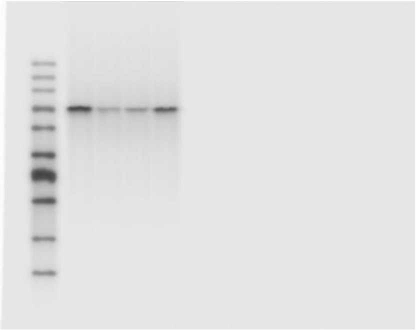

mTOR

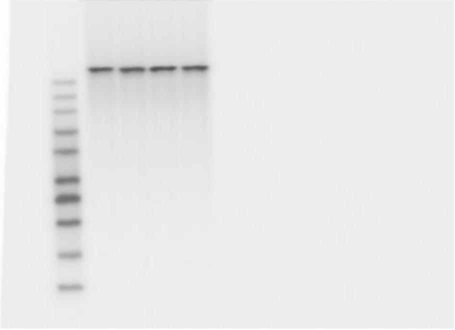

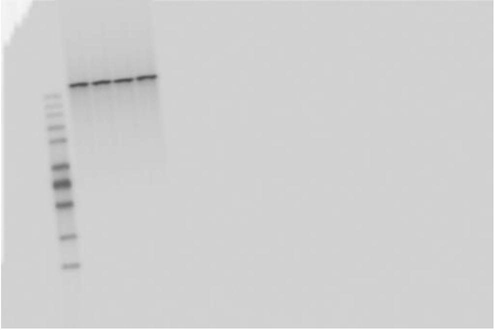

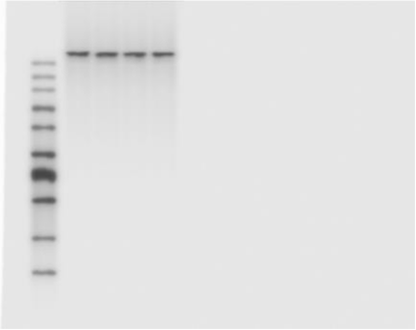

p-mTOR

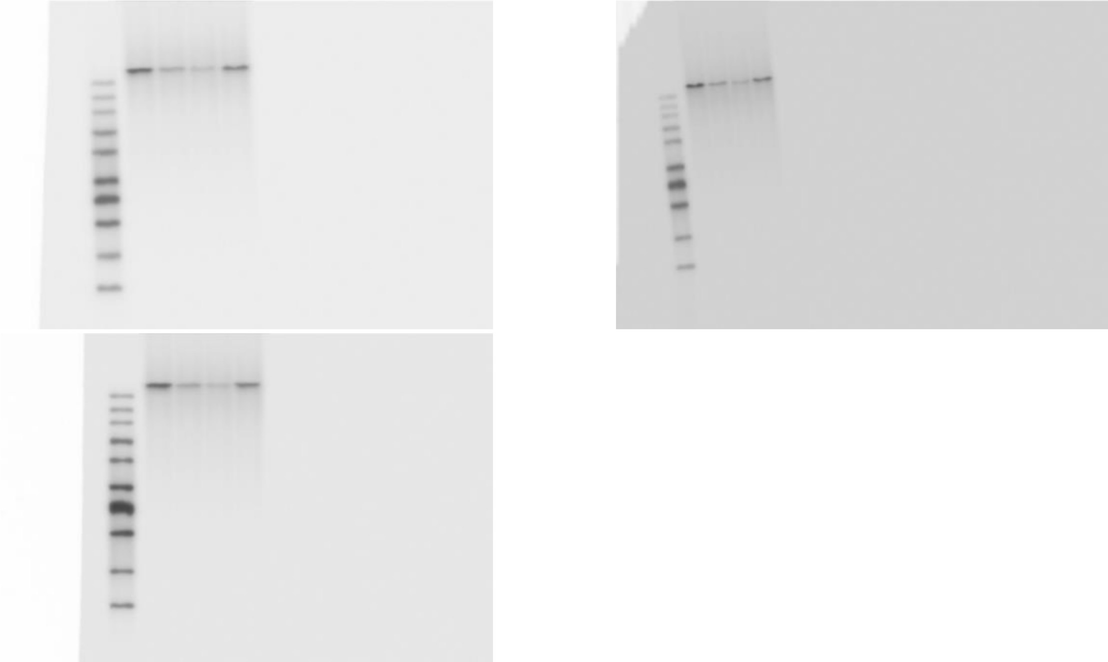

Beclin1

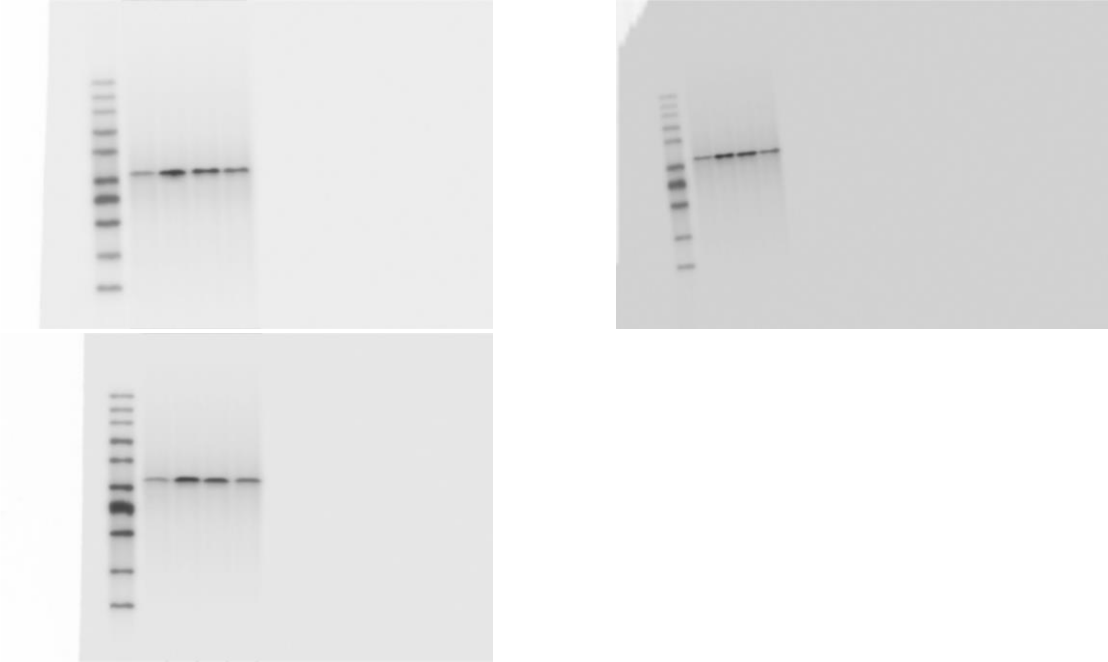

LC3

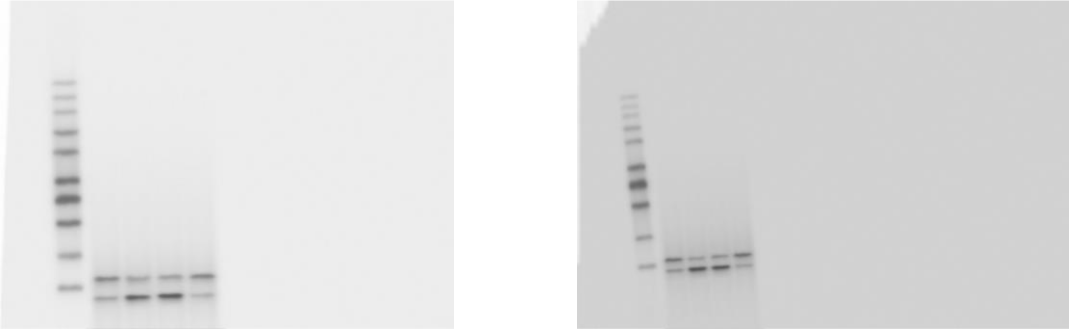

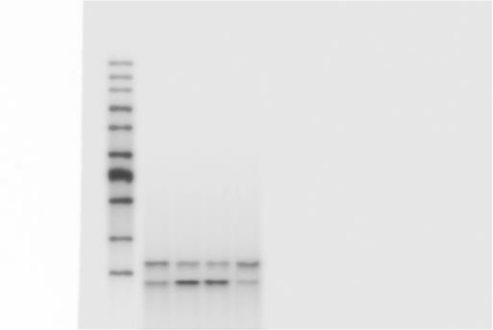

Sox8

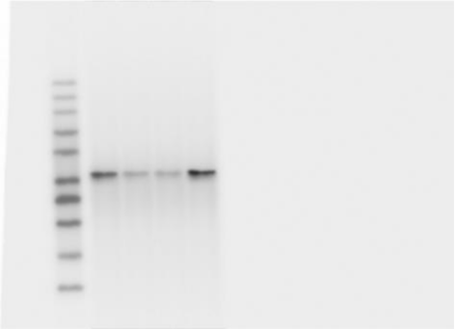

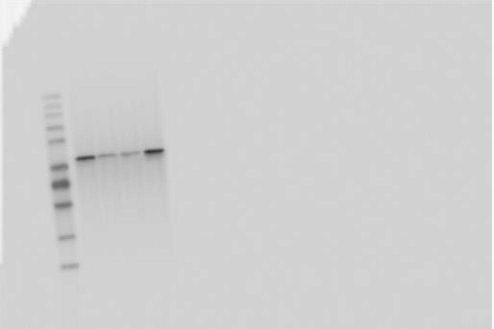

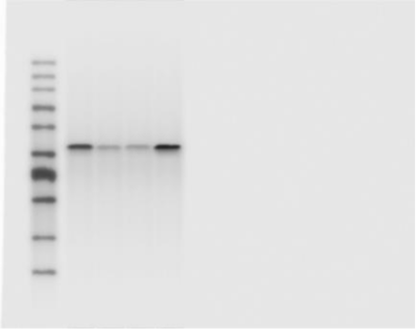

βactin

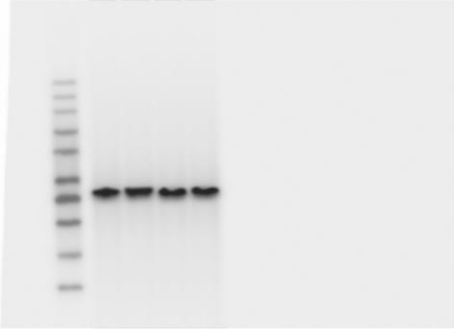

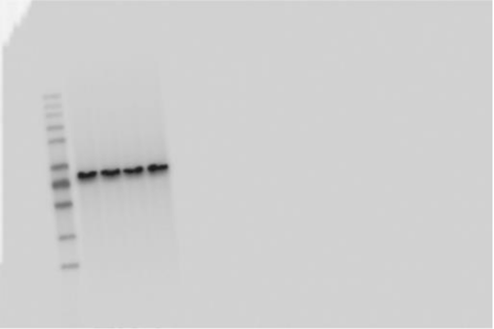

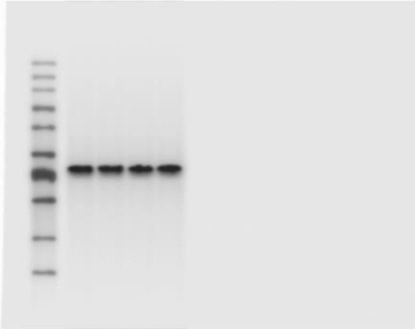

Supplement: Supplementary file 1 — Original Data File [file 41420_2023_1388_MOESM1_ESM.pdf]
