# Supplementary material for: Downregulation of Sox8 mediates monosodium urate crystal-induced autophagic impairment of cartilage in gout arthritis
Source: Cell Death Discov. 2023 Mar 14;9:95. doi: 10.1038/s41420-023-01388-z (PMC10015026; doi:10.1038/s41420-023-01388-z)
Supplement: Supplementary file 2 — supplementary material legends [file 41420_2023_1388_MOESM2_ESM.docx]

**Supplementary figure legends**

**Supplementary Figure 1 Overall flowchart of this study**

**Supplementary Figure 2** **Transfection efficiency of Sox8 overexpression in C28/I2 cells verified by Western blot assay**

Results are the mean ± SD for three individual experiments. *P<0.05, **P<0.01

**Supplementary Figure 3** **Transfection efficiency of silencing of Sox8 in C28/I2 cells verified by PCR**
