# Supplementary figures and images for: Downregulation of Sox8 mediates monosodium urate crystal-induced autophagic impairment of cartilage in gout arthritis
Source: Cell Death Discov. 2023 Mar 14;9:95. doi: 10.1038/s41420-023-01388-z (PMC10015026; doi:10.1038/s41420-023-01388-z)

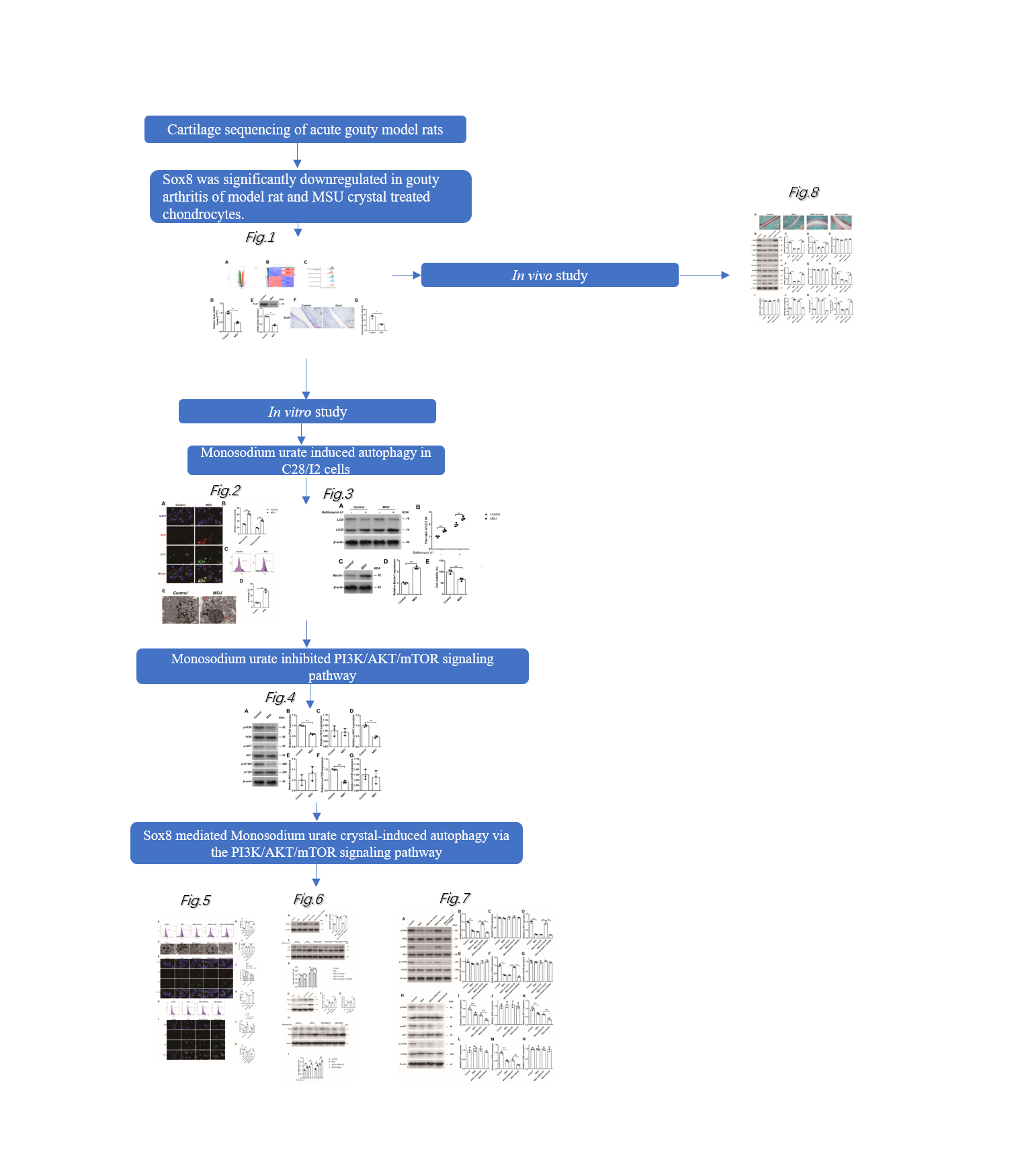

Supplement: Supplementary file 3 — Supplementary Figure 1 [file 41420_2023_1388_MOESM3_ESM.tif]

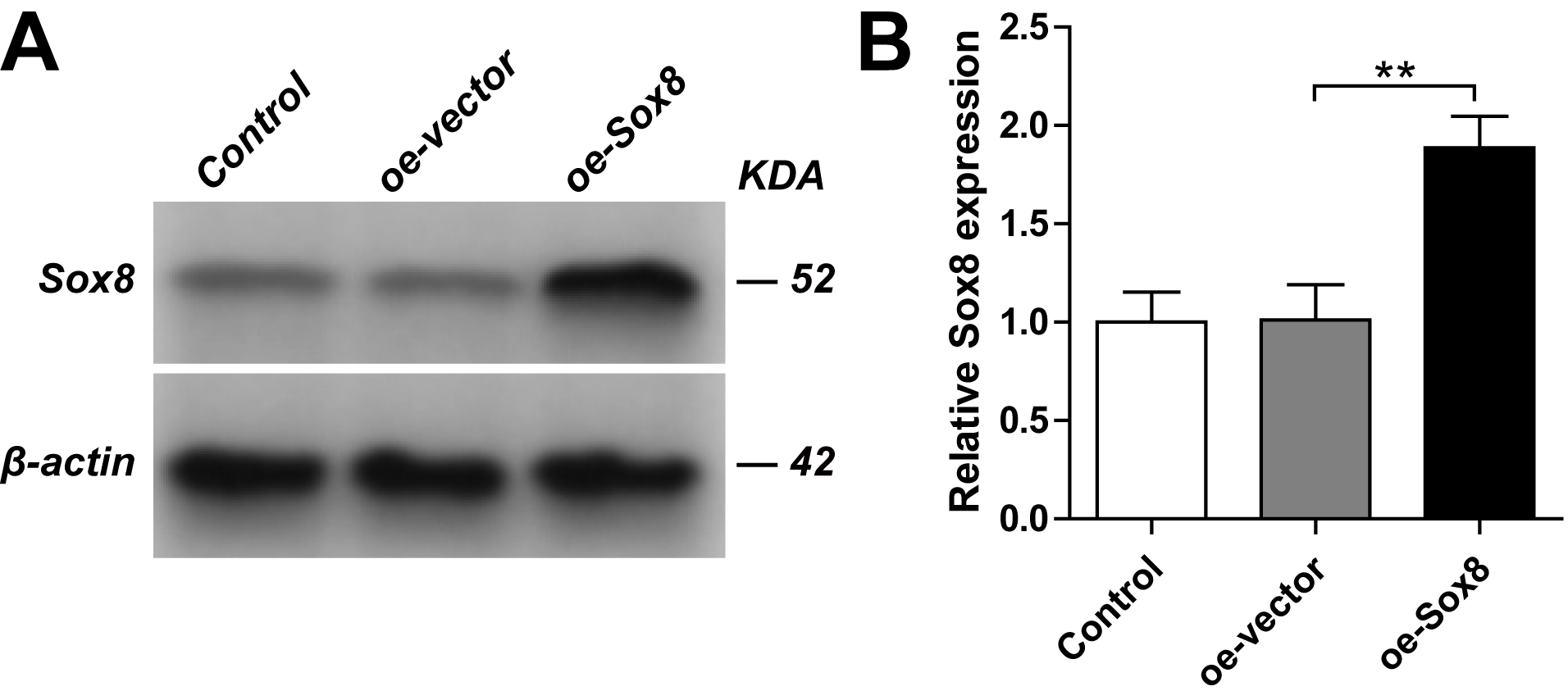

Supplement: Supplementary file 4 — Supplementary Figure 2 [file 41420_2023_1388_MOESM4_ESM.tif]

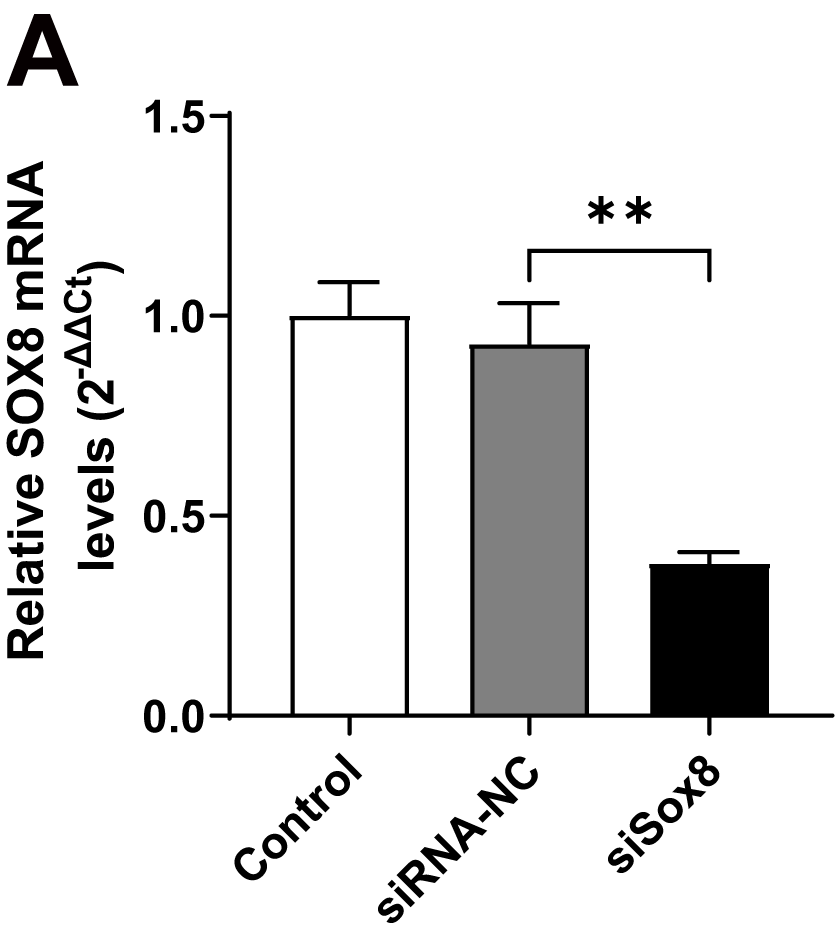

Supplement: Supplementary file 5 — Supplementary Figure 3 [file 41420_2023_1388_MOESM5_ESM.tif]
